# Automated Recognition of *Plasmodium falciparum* Parasites from Portable Blood Levitation Imaging

**DOI:** 10.1002/advs.202105396

**Published:** 2022-08-11

**Authors:** Shreya S. Deshmukh, Oswald Byaruhanga, Patrick Tumwebaze, Demir Akin, Bryan Greenhouse, Elizabeth S. Egan, Utkan Demirci

**Affiliations:** ^1^ Department of Bioengineering Stanford University Schools of Engineering and Medicine Stanford CA 94305 USA; ^2^ Canary Center for Early Cancer Detection Bioacoustic MEMS in Medicine Lab Department of Radiology Stanford University School of Medicine Palo Alto CA 94305 USA; ^3^ Infectious Diseases Research Collaboration Kampala Uganda; ^4^ Department of Medicine University of California San Francisco San Francisco CA 94110 USA; ^5^ Department of Pediatrics Stanford University School of Medicine Stanford CA 94305 USA; ^6^ Department of Microbiology and Immunology Stanford University School of Medicine Stanford CA 94305 USA

**Keywords:** computer vision, malaria, portable imaging, resource‐limited settings

## Abstract

In many malaria‐endemic regions, current detection tools are inadequate in diagnostic accuracy and accessibility. To meet the need for direct, phenotypic, and automated malaria parasite detection in field settings, a portable platform to process, image, and analyze whole blood to detect *Plasmodium falciparum* parasites, is developed. The liberated parasites from lysed red blood cells suspended in a magnetic field are accurately detected using this cellphone‐interfaced, battery‐operated imaging platform. A validation study is conducted at Ugandan clinics, processing 45 malaria‐negative and 36 malaria‐positive clinical samples without external infrastructure. Texture and morphology features are extracted from the sample images, and a random forest classifier is trained to assess infection status, achieving 100% sensitivity and 91% specificity against gold‐standard measurements (microscopy and polymerase chain reaction), and limit of detection of 31 parasites per µL. This rapid and user‐friendly platform enables portable parasite detection and can support malaria diagnostics, surveillance, and research in resource‐constrained environments.

## Graphical abstract

This study presents an automated phenotypic approach to detecting malaria parasites in field settings. Red blood cells are lysed then suspended in a magnetic field, to be imaged on a portable imaging platform. Morphological and texture features are extracted from this image data and used to train a classifier with 95% accuracy on patient samples tested in eastern Uganda.

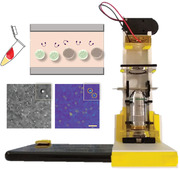

## 1 Introduction

A significant barrier to effective healthcare in regions with infrastructure limitations is access to appropriate diagnostics. Novel approaches to disease detection have ranged from adapting existing technologies for different settings (e.g., radiological imaging^[^ ^1^, ^2^ ^]^ or sequencing^[^ ^3^, ^4^ ^]^), to transferring systems to new applications (e.g., repurposing an Human Immunodeficiency Virus and Acquired Immunodeficiency Syndrome (HIV/AIDS), contact tracing system for coronavirus disease 2019 (Covid‐19)^[^ ^5^ ^]^), to out‐of‐the‐box innovations (e.g., an acetic acid test for cervical cancer^[^ ^6^ ^]^). Such varied approaches can circumvent the significant resource barriers at the root of the higher morbidity and mortality in economically challenged regions.^[^ ^7^, ^8^ ^]^ While tradeoffs are often made between diagnostic performance and accessibility factors (e.g., cost), innovative solutions and human‐centered designs can minimize cost and infrastructure requirements while still upholding high healthcare standards and instituting locally appropriate measures.^[^ ^9^ ^]^ Such approaches spur diagnostic innovations that act across a range of biological scales, employ natural phenomena, and incorporate the latest digital technologies in imaging, data analysis, and data transfer security.^[^ ^10^ ^]^ These tools should also embody the World Health Organization (WHO) standard criteria of “ASSURED (Affordable, Sensitive, Specific, User‐friendly, Rapid and robust, Equipment‐free and Deliverable to end‐users)” for effective diagnostic tests in resource‐limited settings.^[^ ^11^ ^]^

Malaria is a mosquito‐borne disease caused by *Plasmodium* genus parasites and leads to multisystem organ failure. It has devastated human lives for millennia, annually infecting over 200 million people and killing over 400 000 patients. *Plasmodium falciparum* (*P. falciparum*) is responsible for most of the mortality, taking the greatest toll (>70% of cases) on children under 5 years old.^[^ ^12^ ^]^ Young children and pregnant women are most prone to severe outcomes due to limited immunity, susceptibility to anemia and cerebral complications, and HIV co‐infection.^[^ ^13^ ^]^ The impact of malaria has a clear geographical pattern today: 99% of infections occur in developing tropical and subtropical regions in the global south. Here, many people experience inconsistent access to important infrastructural factors for effective counter‐measures that enabled the eradication of the disease elsewhere. These factors can include electricity, specialized medical training, effective mosquito control, and affordable tests.^[^ ^12^ ^]^ Thus, healthcare workers in these settings face obstacles in providing a rapid, accurate, and affordable diagnosis and initiating the appropriate treatment course.^[^ ^14^, ^15^ ^]^ There is a need for easy‐to‐use, cost‐effective, and accurate tools for malaria diagnosis, surveillance, treatment, and management that can be performed within resource‐constrained environments. To meet WHO's target of a 90% reduction in the malaria burden by 2030,^[^^]^ we need affordable, widely available, robust, and quantitative devices at the point of care (POC) that are amenable to scale‐up and cost reduction.

**Figure 1:**
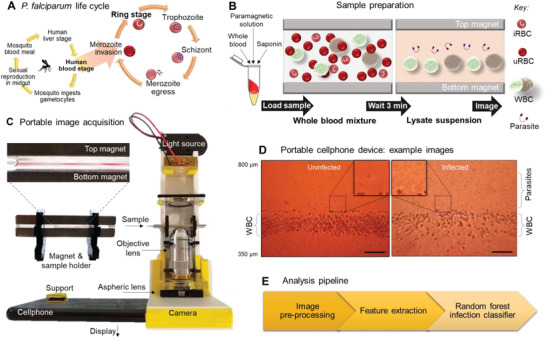
Rapid sample suspension and portable imaging approach for parasite detection. A) Schematic depicting the life cycle of the parasite *Plasmodium falciparum*, describing the stages of the asexual blood‐stage forms, where the ring‐stage parasite is the focus of clinical detection. B) Schematic describing mechanism of sample preparation in levitation chamber. A 10 µL drop of whole blood is mixed with a temperature‐stable solution (saponin and paramagnetic ions in phosphate buffered saline (PBS)) and loaded into a square glass capillary (1 µm tall). The contents are suspended for 3 min, in which the red blood cells (RBCs) lyse to release free hemoglobin and, if infected, intracellular parasites, and all objects in suspension (including unlysed white blood cells (WBCs)) come to equilibrium positions. C) Custom prototype for portable imaging setup of levitated blood samples. Sample capillary is inserted as indicated, backlit with a white light emmitting device (LED), for brightfield imaging, and the magnified and corrected image is viewed on the smartphone screen as captured on its camera. This intuitive display and touchscreen can be used to capture images and focus can be adjusted. The modular design allows for changing magnification and adding fluorescence imaging. D) Example images of whole blood samples from human donors as captured on the portable imaging platform, marked with regions where WBC are present, and where parasites are present: (i) an example of a malaria‐negative blood sample, and (ii) an example of a malaria‐positive blood sample. Scale bar = 100 µm. E) Image analysis and classification workflow summary. Abbreviations:  iRBCs = infected RBCs, uRBCs = uninfected RBCs.

Upon infection from a mosquito bite, *P. falciparum* initially infects and replicates in the liver in a clinically silent manner, and then new merozoites are introduced into the blood stream where they infect erythrocytes. The intra‐erythrocytic cycle lasts 48 h in an individual cell, during which time the parasite induces significant biophysical changes as it matures, and exports virulence proteins to the erythrocyte plasma membrane (as depicted in **Figure** **1**A). These transformations lead to sequestration of later‐stage infected cells (trophozoites and schizonts) within the blood vasculature and organs, primarily due to adherence to host receptors through the *P. falciparum* surface‐expressed proteins and loss of deformability.^[^ ^17^, ^18^, ^19^, ^20^ ^]^ The symptoms of severe malaria are caused by the sequestration of these late‐stage infected cells in capillaries, the disruption of erythrocyte function due to lysis of erythrocytes, and organ damage from the accumulation of cytokines and impaired blood flow.^[^ ^13^ ^]^ Thus, detection of malaria from peripheral blood samples depends on identifying the earliest stage of the parasite, the ring, as this is the only stage found in circulation.^[^ ^21^, ^22^ ^]^ Direct detection of this 1–3 µm parasite structure inside the 5–7 µm‐wide host erythrocyte requires exceptional assay sensitivity, whether by visual, biophysical, or molecular means. This is made more challenging in the resource‐limited conditions of most malaria‐endemic regions.^[^ ^23^ ^]^ Among field‐appropriate techniques, Giemsa‐stain microscopy uses visual morphological examination by an expert to detect infected erythrocytes in the ring stage. While inexpensive and widely implemented, this technique is time‐consuming and technically demanding, especially when parasite densities are low, because it requires manual analysis of individual erythrocytes. The process of examining a sufficient number of fields on a microscope slide to assess the infection status of each erythrocyte is highly time‐consuming. Hence, malaria infections with low parasitemia (<200 parasites per µL) are often missed by microscopic evaluation.^[^ ^24^ ^]^ Since parasitemia fluctuates seasonally (with wet weather corresponding with subsequently higher malaria transmission), this sensitivity is particularly crucial in dry seasons when parasitemia tends to be lower.^[^ ^25^ ^]^ The other limitations of microscopic evaluations include the inherent user‐to‐user variability and the requirement for cell fixation/staining, which bars most downstream analyses. Antibody‐based lateral flow rapid diagnostic tests (RDTs) have demonstrated great success in usability and achieving a rapid sample‐to‐answer turnaround time (≈20 min).^[^ ^26^ ^]^ The RDTs detect histidine‐rich protein 2 or 3 (Pf‐HRP2/3) for *P. falciparum*, and *Plasmodium* lactate dehydrogenase or aldolase for other strains.^[^ ^14^, ^27^ ^]^ The Pf‐HRP2 tests are preferred for greater sensitivity and thermal stability, especially in African regions where *P. falciparum* is dominant.^[^ ^28^ ^]^ However, it is difficult to practically evaluate RDTs' effectiveness when stored under inconsistent temperatures or humidity settings.^[^ ^29^, ^30^, ^31^ ^]^ The other limitations of these assays include the risk of false‐negative results (thus reduced sensitivity) due to reported pfhrp2,3 deletions (in laboratory and field isolates),^[^ ^32^, ^33^, ^34^ ^]^ and the risk of false‐positive results (thus reduced specificity) since the antigens can persist in circulation for over 4 weeks after the infection has resolved.^[^ ^35^ ^]^ Further, antigen‐detecting tests generally lack the capability to quantify other parameters such as drug susceptibility. Beyond these techniques, polymerase chain reaction (PCR)‐based tests for malaria have superior sensitivity and specificity with limits of detection below ten parasites per µL, and often act as gold standard references to evaluate other diagnostic methods.^[^ ^36^, ^37^, ^38^ ^]^ However, due to its relatively high dependence on laboratory infrastructure, it is not routinely used outside of epidemiological studies, or species identification after initial microscopy‐ or RDT‐based diagnosis.^[^ ^37^, ^39^ ^]^

As summarized above, while various malaria detection platforms are available for POC testing, there is a significant unmet need for a field‐appropriate POC tool with the capabilities of sensitive, specific, and quantitative detection for malaria infections.^[^ ^8^, ^40^ ^]^ To address this need, we demonstrate a portable POC detection platform to assess malaria infections from clinical whole blood samples. Combining chemistry, biophysics, optics, and machine learning, we designed an inexpensive, easy to operate, battery‐operated device that can rapidly detect parasites in blood and perform dynamic, quantitative measurements outside of laboratory environments. Using saponin (a widely available and temperature‐stable compound) for lysis of erythrocytes enabled direct measurements of intracellular parasites and improved the signal‐to‐noise ratio by rendering erythrocytes optically clear. To identify the *P. falciparum* parasites, we lyse whole blood and suspend the lysate in a paramagnetic fluid that separates the erythrocyte components and the parasites for the high spatial resolution measurement. We employ increasingly ubiquitous smartphone technology^[^ ^41^ ^]^ to design an inexpensive, compact, and battery‐operated microscopic imaging setup to measure magnetically levitated blood sample lysates. We combine these measurements with machine learning to enable rapid and accurate morphological classification of samples, so that this approach is readily available regardless of the local infrastructure constraints.

## 2 Results

### 2.1 Approach

We designed this platform to perform accurate and quantitative detection of *P. falciparum* parasites from whole blood clinical samples in field settings. To optimize for sensitive detection, we first pursued a strategy to liberate *P. falciparum* parasites from erythrocytes using saponin lysis, followed by magnetic levitation to separate the constituents of the blood lysate. Then, we developed a custom smartphone‐based platform to achieve sample imaging and parasite detection in an inexpensive and portable format. Finally, we developed a random forest classifier for automated sample analysis, validating its performance in a pilot study conducted with clinical whole blood samples collected on‐site in three regions of eastern Uganda; Busia, Nagongera, and Tororo.

Figure 1 depicts the overall approach, starting with the sample preparation workflow in Figure 1B. The suspension consists of a finger‐prick drop‐sized volume (10 µL) of whole blood mixed with the test solution containing saponin for lysis and gadolinium for magnetic levitation. This mixture is loaded into a 1 mm square glass capillary, inserted into the space between two neodymium magnets, as in Figure 1C. Within 3 min, the erythrocytes lyse, and the individual components come to equilibrium positions in the magnetic field, without agitation, centrifugation, or filtration. The process of erythrocyte lysis releases free hemoglobin and parasites that are distinguishable from leukocytes (which remain primarily unlysed) due to their distinct size, morphology, and levitation positions. This lysed and suspended sample is imaged at equilibrium in the imaging platform, as shown in Figure 1C. The loaded sample capillary is inserted as indicated and illuminated for magnified display on the smartphone. This modular design permits magnification adjustment and adding fluorescence imaging capability per the specific imaging needs of the individual test. The images of suspended blood components (e.g., in Figure 1D) could be used in a range of analysis pipelines, from direct visual confirmation of parasites to the extraction and quantification of more specific morphological features (e.g., shape and texture). We extracted a set of specific morphological features from the images to train an automated random forest classifier for sample images, as in Figure 1E.

The presented system is a unique combination of rapid sample processing coupled with direct visual detection and accurate analysis of parasites. This system and the workflow enable inexpensive quantification of the malaria parasites from the blood samples within minutes with an approximate cost of $1 per test and $30 for the portable reusable imaging platform (Figure S1A, Supporting Information), in a form factor using approximately one square foot of tabletop area and entirely battery‐operated components (Figure S1B, Supporting Information).

### 2.2 Investigation of Direct Ex Situ Parasite Detection via Whole Blood Lysis

**Figure 2:**
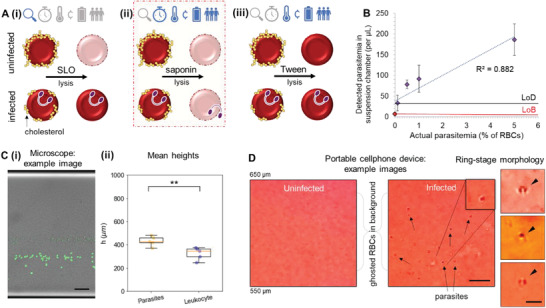
Investigating erythrocyte lysis to aid direct visual parasite detection. A) Different approaches to lysis of RBCs for detection of parasites, with the symbols above indicating suitability for resource‐limited settings, as discussed in the text: (i) Streptolysin O, (ii) saponin, (iii) Tween 20. B) Dilution of culture‐grown *P. falciparum* under saponin lysis (*n* = 3 blank samples, *n* = 10 parasite‐containing samples) in levitation suspensions at 50 mm of gadolinium, to determine the limit of detection (31 parasites per µL). Actual parasitemia is calculated from the known parasitemia of the culture and the dilution factor, while detected parasitemia is calculated from the concentration of parasites detected visually in each volumetric image field. Detected parasitemia correlated to actual parasitemia with *R* ^2^ = 0.882. C) (i) Example microscope image of whole blood spiked with cultured parasites, subject to the saponin lysis protocol. Acridine orange stains nucleic acids green, thus highlighting the two bands of parasites (released from RBCs) and WBCs (unlysed) respectively. (ii) The box‐and‐whisker plot shows the mean height of bands in samples of lysed parasites from culture, that is, parasite bands (*n* = 5) and lysed uninfected whole blood, that is, leukocyte bands (*n* = 5). Both groups were significantly different under a *t*‐test with a *p*‐value of 0.0089 (two asterisks). Each element in the box plot represents the following: center line represents the median; box limits represent the upper and lower quartiles; whiskers represent the range of the data. D) We selected the saponin lysis protocol to use with malaria‐infected and uninfected human whole blood samples. Close‐up images of saponin‐lysed whole blood samples from human donors, as captured on the portable imaging platform, to show cell and lysate structures in more detail: (left) an example of a malaria‐negative blood sample, and (middle) an example of a malaria‐positive blood sample. The orange–red background color of the solution is attributed to free hemoglobin from lysed RBCs, the light mottled pattern in the background is attributed to ghosted RBC membranes, and the small dark circular structures in the foreground are attributed to the intracellular ring‐stage parasites. Scale bar = 10 µm. (Far right): higher‐zoom images showing examples of ring‐stage morphology. Scale bar = 5 µm. Abbreviations: RBCs = red blood cells, WBC = white blood cells. Symbols in (A), from left to right: sensitive, rapid, temperature‐stable, low‐cost, electricity‐independent, user‐friendly.

We have previously published a levitation‐based biophysical manipulation system for the stage‐wise separation of *P. falciparum*‐infected erythrocytes.^[^ ^42^ ^]^ To improve the applicability of this system for clinical whole blood samples in field settings, we considered various adaptations to improve the usability and accuracy of this original system. First, we investigated approaches to detect parasites in cultured samples under laboratory settings before working with clinical samples from field sites. We pursued an erythrocyte lysis approach to improve the compatibility of our system with clinical whole blood samples, which may have lower parasitemia and contain more complex blood components (e.g., leukocytes) than in vitro cultured erythrocytes. Further, lysis would enable direct visual detection and improve accuracy by eliminating uninfected erythrocytes from the visual field (since they resemble ring‐stage infected erythrocytes). While there are many ways to lyse erythrocytes, we focused on the modes of lysis that differentially bind to the cholesterol in erythrocyte membranes. Since parasites recruit excess cholesterol from the host erythrocyte membrane during their development, infected erythrocyte membranes are left with depleted cholesterol levels than uninfected erythrocytes.^[^ ^43^, ^44^ ^]^ The use of detergents allows for selective lysis of erythrocyte membranes without disrupting intracellular parasitic membranes.^[^ ^44^, ^45^, ^46^ ^]^ We investigated the lysis efficacy of three modes of detergent‐like lysis methods: streptolysin O (SLO), saponin, and Tween‐20, as well as the suitability of each method for practical use in resource‐limited settings, as compared in **Figure** **2**A. While SLO can selectively lyse uninfected cells quickly, the lysis is inconsistent; furthermore, the protein is relatively unstable, requiring refrigerated storage and temperature‐controlled activation steps (Figure 2A[i]). While Tween‐20 is more temperature‐stable, it achieves lysis too slowly to be suitable for a rapid technique (Figure 2A[iii]). Saponin is also of biological origin, widely available, and lyses all erythrocytes. The lysis by saponin is rapid and the reaction is temperature‐stable at the intended application settings; consequently, saponin can be implemented with basic techniques without requiring any additional equipment (Figure 2A[ii]).

Based on the described characteristics, we pursued a saponin‐based lysis approach. To investigate the sensitivity of this lysis‐based detection strategy, we performed serial dilutions of laboratory‐adapted *P. falciparum* cultured in human erythrocytes, then induced saponin lysis in our levitation suspensions to determine a theoretical limit of detection. Actual parasitemia was calculated by the standard procedure (enumerating parasitemia on a blood smear by Miller reticle) and its subsequent dilution factor. Detected parasitemia was quantified by the number of released parasites assessed by our visual detection method that uses an object‐counting algorithm (described in Experimental Section) in each image field of the levitation chamber. As Figure 2B shows, there was a strong correlation between actual and detected parasitemia levels (*R* ^2^ = 0.882).^[^ ^47^ ^]^ These results indicate that parasites can be detected in a lysed suspension, at least under laboratory conditions. Thus, we selected the saponin lysis protocol for our subsequent analysis of whole blood samples since this degree of lysis supported sensitive and specific detection and seemed suitable for our targeted application area in resource‐limited settings.

To determine the sensitivity of our approach in distinguishing parasites from the other components of whole blood, we focused on optimizing the separation of parasites from leukocytes in whole blood. We investigated this by spiking cultured parasites into uninfected whole blood from donors, in which leukocytes and erythrocytes showed a levitation height separation profile as shown in Figure 2C as well as differentiable detected object radii. Further optimization, as shown in Figure S2, Supporting Information, suggested that a higher magnetic susceptibility of the levitation medium (by increasing the concentration of paramagnetic ions) showed better concentration into individual bands and therefore improved separation, as well as a faster time‐to‐result. In magnetic levitation, increasing the medium magnetic susceptibility also increases the velocity at which objects travel to their final equilibrium height where effective forces on the objects are balanced. Further, smaller elements (e.g., the liberated intracellular parasites) travel more slowly than larger elements (e.g., leukocytes) and therefore benefit from a more concentrated paramagnetic medium for timely arrival to their equilibrium positions.^[^ ^48^ ^]^

Testing the lysis protocol with parasites from clinical samples showed distinct visual morphologies that match the known and observed morphology from culture samples, as can be seen in Figure S3, Supporting Information. Close‐up images of saponin‐lysed whole blood samples from malaria‐positive human donors, captured on the portable imaging platform (in Figure 2D), show the cell and lysate structures in more detail. The orange–red background color of the solution comes from free hemoglobin from lysed erythrocytes, the light mottled background pattern from ghosted erythrocyte membranes, and the small dark circular structures from the intracellular ring‐stage parasites.

### 2.3 Portable Cellphone‐Based Imaging of Cells in Levitation

We designed a custom prototype for portable imaging of levitated blood samples, as pictured in Figure 1C. The sample capillary is inserted as indicated, backlit by a light‐emitting diode (LED) for brightfield imaging, and the magnified and corrected image is viewed on the smartphone screen as captured on its camera. The intuitive touchscreen is used to view the sample in real time, adjust focus and zoom, and capture images for subsequent analysis. The complete optical path is described in Figure S1A, Supporting Information.

First, a coin battery powers a white LED in a laser‐cut plastic holder. Next, another laser‐cut sample holder carries the magnets and the loaded capillary. To multiplex sample analysis, individual holders can be replaced to image multiple samples in quick succession. Adjacent to the sample window, we use a 20× objective lens for magnification. Subsequently, we perform aberration correction with an aspheric lens. Finally, we attach a camera‐enabled cellphone (which provides a lens, sensor, and screen in a Redmi model as detailed in the Experimental Section) in a 3D‐printed removable phone holder that interfaces with the main 3D‐printed body of the imaging platform. Other smartphone models that include cameras of equivalent resolution or higher could also potentially be interfaced with this setup by using custom‐printed phone holders. Optional inserts for emission and excitation filters can be added for fluorescence imaging.

### 2.4 Clinical Sample Acquisition and Analysis in Malaria‐Endemic Field Settings

We imaged cells at their equilibrium height positions in our cellphone‐based imaging system and performed image analysis using a custom‐written Python algorithm to distinguish the properties of different cell populations based on their visual image attributes. The analysis workflow is described in Figure S4, Supporting Information, with further details in the Experimental Section.

Whole venous blood samples were obtained for processing and data acquisition, drawn into heparinized tubes, and subsequently processed on‐site at three locations in eastern Uganda: Tororo District Hospital, Masafu General Hospital (Busia), and Nagongera Health Center IV. These studies were conducted through a partnership with the Infectious Diseases Research Collaboration‐Uganda, using small unused volumes of blood samples collected through their ongoing approved clinical studies. The samples we received were de‐identified of any information beyond parasitemia. These samples included both malaria‐negative subjects and malaria‐positive patients who met the study criteria (of note, with parasitemia levels = greater than 1% as measured by smear microscopy). We used our optimized protocol for blood lysis and suspension on 10 µL of each whole blood sample and captured images as described above for subsequent analysis. We did not use external equipment or electricity during sample processing and data capture other than charging the smartphone approximately once per week. Example images from malaria‐negative and positive whole blood samples can be found in Figure 1D, as captured on the portable imaging platform.

### 2.5 Quantification of Cellular Elements in the Blood of Malaria Patients

**Figure 3:**
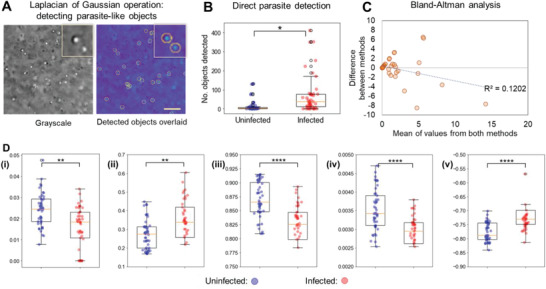
Automated image processing to extract visual features associated with parasites. A) A Laplacian‐of‐Gaussian (LoG) blob detection algorithm developed to directly and specifically detect the ring‐stage parasites, and differentiate them from other structures (the background, which can contain ghosted RBC membranes, as well as intact WBCs which are larger and of a different shape). Scale bar = 10 µm. B) The results of the LoG algorithm to quantify parasite structures in the patient sample images acquired on the portable platform. The quantification is the number of blobs detected per sample image. Performing a z‐test on both groups resulted in a test statistic of −2.2 and a *p*‐value of 0.025 (marked with one asterisk). Using a threshold of 10, sample images can be classified into negative (below 10) or positive (greater than or equal to 10). C) A Bland–Altman analysis was conducted to compare the parasitemia quantification accuracy of this algorithm to the gold standard method of expert‐examined Giemsa‐stained smear microscopy. D) Image analysis of texture features was performed on gray‐level co‐occurrence matrixes (GLCM) of the image pixels, using Haralick's method to extract 13 specific features calculated from the relationships between pixel structures in the GLCM. These features were calculated, per image, for four directions each, and the difference therein between malaria‐negative and malaria‐positive samples was explored. The directional means of the five features that contained the most predictive differences between the two groups are presented here: (i) angular second moment (test statistic: 2.7, *p*‐value: 0.0076), (ii) contrast (test statistic: −3.1, *p*‐value: 0.0020), (iii) inverse difference moment (test statistic: 4.4, *p*‐value: 0.000013), (iv) difference variance (test statistic: 4.5, *p*‐value: 0.0000057), and (v) first informational measure of correlation (test statistic: −4.9, *p*‐value: 0.00000076). Notes on the box‐and‐whisker plots: Each element represents the following: center line represents the median; box limits represent the upper and lower quartiles; whiskers represent the range of the data, circles represent outliers in the data. Abbreviations: RBCs = red blood cells, WBC = white blood cells.

We pursued a machine learning approach trained on specific morphology features for optimal classification accuracy with the patient sample image data and compatibility with a relatively small dataset. **Figure** **3**A shows example images of lysed whole blood patient samples from the portable imaging setup. Examples of liberated parasites and intact white blood cells (WBCs) are labeled on these images to illustrate the morphologies we encounter. We optimized an object detection method to directly and specifically detect the ring‐stage parasites and differentiate them from other structures (the background containing ghosted RBC membranes, platelets, and intact leukocytes, which are larger and of a distinct shape), as labeled in Figure S5, Supporting Information. For this, we implemented a Laplacian‐of‐Gaussian (LoG) convolution, which is commonly applied to detect cells and cellular structures in biomedical images, among other blob detection applications.^[^ ^49^, ^50^ ^]^ We document the performance of the LoG algorithm in quantifying parasite‐like structures in the patient sample images acquired on the portable platform in Figure 3B. We found the numbers of detected parasite‐like objects to be different between malaria‐negative and malaria‐positive groups, with a *p*‐value of 0.02. We tested a range of LoG parameters (threshold, kernel maximum, and step size) to determine the combination that led to optimal accuracy at parasite detection, as shown in Figure S6, Supporting Information.

### 2.6 Comparison to Gold Standard *Plasmodium* Detection Methods

In Figure 3C, a Bland–Altman analysis was conducted to compare the parasitemia quantification accuracy of the LoG object detection algorithm to the gold‐standard method of expert‐examined Giemsa‐stained smear microscopy. Since patient samples were collected from multiple sources and validated by either microscopy or qPCR, the results displayed here are only those validated by smear microscopy to provide a single comparison. These were primarily malaria‐positive samples with a parasitemia of 1% or higher due to the study criteria. We found the parasitemia to be generally correlated, but with an underestimation trend as parasitemia level increased.

### 2.7 Descriptive Morphological Features in the Lysate to Detect the Presence of *Plasmodium* Parasites

We quantified various parameters of the textures observed in sample images by calculating the gray‐level co‐occurrence matrix (GLCM), an approach used since 1970 for medical cell image applications.^[^ ^51^ ^]^ We pursued this approach for its invariance to gray tone (pixel intensity) calibration, which can be challenging to keep consistent in cellphone‐based images with slightly varying ambient illumination. While it has limitations in capturing the large shape‐based features, this is well‐suited for our images where we aim to discern fine‐grained features (corresponding to the parasitic bodies), and we aim to avoid bias from coarser features that are likely to be from non‐parasitic whole blood components or debris.^[^ ^52^ ^]^ We used Haralick's method to extract 13 features that describe the tonal primitive properties of an image and its spatial organization via pixel interrelationships.^[^ ^53^ ^]^ Each feature is calculated in all four directions present in the 2D images (horizontal, vertical, left diagonal, right diagonal, symmetric across each of those directions respectively, i.e., the horizontal direction is computed for left‐right and right‐left directions). These features were calculated per image, and the differences between malaria‐negative and malaria‐positive samples were examined for separability between malaria‐negative and positive groups (first as the mean across the four directions, for rotational invariance). We show in Figure 3D the five features that seem most separable based on a z‐test: 1) angular second moment (sum of squares, a measure of uniformity), 2) contrast (weighted toward dissimilar neighbors more than similar neighbors, a measure of local variance), 3) inverse difference moment (a measure of homogeneity), 4) difference variance, and 5) first informational measure of correlation (how well pixels correlated to their neighborhoods).^[^ ^54^, ^55^ ^]^ The same data for all 13 features are presented in Figures S7–S9, Supporting Information. After the preliminary comparison in Figure 3D, we next considered the texture features in each direction as independent features. While there is a class separation between the groups in some features, no single feature is separable enough to be used as a classifier on its own.

### 2.8 Using Machine Learning for Infection Status Classification with a Random Forest Model

Principal components analysis, an unsupervised classification approach, as shown in Figure S10, Supporting Information, does not independently demonstrate clear enough class separation. Thus, we looked to other machine learning techniques applicable to the specific features we extracted. The machine learning field has benefited from recent computational and algorithmic improvements, becoming more accessible and less computationally costly. These techniques have recently been applied to many medical image data problems, including classification. We applied various classical machine learning models from Python's scikit‐learn module to our dataset, initially with default hyperparameters, to estimate which models might be most suitable for this task, as shown in Figure S11, Supporting Information. We proceeded to tune the hyperparameters of the most promising models from this initial search, selecting approaches known to perform well in similar tasks (i.e., medical image‐based datasets of small size): decision trees and random forest classifiers (ensembles of decision trees).^[^ ^56^, ^57^, ^58^ ^]^ We used the “GridSearchCV” function to systematically search the hyperparameter space for each of these models and used cross‐fold validation to compare the optimized classification performance of each to avoid overfitting with the small dataset. We proceeded with a random forest classifier model, which showed superior performance in this preliminary search, and reduced the risk of overfitting due to the use of bagging and partial feature selection in each tree by design.^[^ ^59^ ^]^

**Figure 4:**
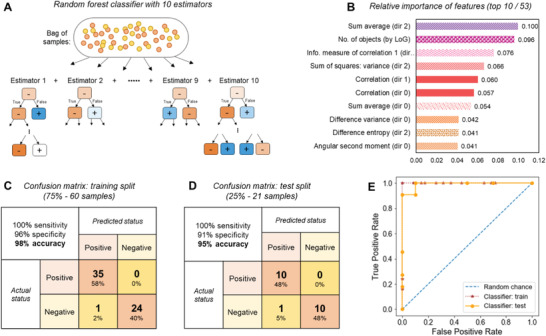
Building a machine learning classifier of infection status using image features. A) A visualization of the random forest classifier applied to our available dataset, which consisted of 53 features (13 Haralick features, each in four directions, and the number of parasite‐like objects detected in each image by the Laplacian‐of‐Gaussian (LoG) blob detection algorithm) for a total of 81 samples, which were randomly split into a training set (75%, or 60 samples) and a test set (25%, or 21 samples). This classifier used ten estimators, all of which can be seen in the supplement. B) Of the 53 features used, the 10 most important are shown here, along with their relative contributions to the performance of the classifier. C) A confusion matrix quantifies the performance of this classifier on the training set, with 100% sensitivity, 96% specificity, and 98% accuracy overall. D) A confusion matrix quantifies the performance of this classifier on the test set, with 100% sensitivity, 91% specificity, and 95% accuracy overall. E) A receiver operating characteristic (ROC) curve is drawn for the classifier, on both training and test sets, with both showing high area under the curve (AUCs) and consistent performance between the training and test set. The training set AUC was 1.00 and the test set AUC was 0.991. Abbreviations: RBCs = red blood cells, WBC = white blood cells.

The dataset consisted of 53 total features (13 Haralick texture features, each in four directions—in case of predictive directional differences—and the parasite‐like object count from the LoG filter), each for 81 samples. These data were randomly split into a training set (75%, i.e., 60 samples) and a test set (25%, i.e., 21 samples) held out during training. A visualization of the random forest classifier applied to our available dataset is shown in **Figure** **4**A. This classifier was implemented in Python using the scikit‐learn module. The model was a meta‐estimator that used ten estimators since the dataset size is relatively small, as pictured in Figure S12, Supporting Information. Bootstrap aggregating used to build each tree, and out‐of‐bag samples were not permitted in training to estimate the generalization accuracy. The choice of parameters for feature selection and error calculation are explained further in the Experimental Section.

A machine learning approach can inform us of more detailed insights into which features are most predictive for classifying blood samples. Calculating the relative feature importance shows the ten most important features, in Figure 4B. The complete list of features and their relative contributions to classification power can be seen in Figure S13, Supporting Information.

The confusion matrix in Figure 4C quantifies the performance of this classifier on the training set, with 100% sensitivity, 96% specificity, and 98% accuracy overall. The confusion matrix in Figure 4D quantifies classification performance on the previously unseen test set, with 100% sensitivity, 91% specificity, and 95% accuracy overall. Within the scope of the data available to us, these results demonstrate good performance on new samples without significant overfitting. Good sensitivity is particularly critical in a diagnostic for treating potential malaria cases. In Figure S14, Supporting Information, a confusion matrix quantifies the performance of this classifier on the full dataset (81 samples in total), with 100% sensitivity, 94% specificity, and 98% accuracy overall. A receiver operating characteristic curve is drawn for the classifier in Figure 4E, on both training and test sets, with both showing high area under the curve and consistent performance between the training and test sets. Thus, our prototype design showed significant promise in this pilot study, with initial validation that our classifier has high accuracy compared to gold‐standard field diagnostics such as Giemsa‐stained smear microscopy by a trained expert, and adequately stored RDTs, even with data collected in “field” conditions, that is, with limited space, and without relying on consistent electricity access, refrigeration, incubation, centrifugation, or other complex infrastructure.

## 3 Discussion

There is a persisting need for a malaria parasite detection strategy that can deliver reliably accurate analyses in varying and often resource‐constrained conditions. Here, we demonstrate an interdisciplinary approach that performs rapid and automated analysis of fresh whole blood samples, generating versatile image data in real time. Our design frees the platform and its user from most infrastructural constraints, increasing its accessibility to many locations beyond traditional laboratories. To investigate the performance and utility of this system, we tested a prototype in multiple such resource‐limited sites in eastern Uganda, validating its results with whole blood samples from malaria patients, along with gold‐standard microscopy and qPCR testing. Following a simple sequence of sample mixing, loading, suspension, imaging, and finally automated analysis, we demonstrate a proof‐of‐concept of achieving rapid and accurate *P. falciparum* parasite detection on a compact, portable, and user‐friendly prototype with entirely battery‐operated components.

Our method relies on a saponin‐based lysis approach to improve the sensitivity of malaria‐causing parasite detection in complex whole blood clinical specimens. This approach also has the advantage of enriching parasites that may be at a low concentration. Uninfected erythrocytes do not differ greatly in size or morphology externally from ring‐stage infected erythrocytes unless fixing and staining are used. Thus, we lysed all erythrocytes and rendered them optically translucent in our imaging to reveal the parasites released from their host erythrocytes. The released ring‐stage parasites are more readily differentiable from the remaining leukocytes, which do not lyse but are larger and morphologically distinct from parasites; the effect of saponin on leukocyte height is documented in Figure S15, Supporting Information. This approach also enabled the use of less diluted blood than would be used in levitation with unlysed blood, allowing us to use 10 µL of whole blood for adequate sensitivity, yet an adequately small volume to be obtained from typical finger‐prick sampling. Thus, our lysis‐based approach improved the signal‐to‐noise ratio in our samples by eliminating erythrocytic bodies and enriching parasites while still being compatible with whole blood drawn from patients.

The lysis and release of parasites enables their direct visual detection, which opens various possibilities for data collection and analyses. This modality can empower a clinician in visually confirming these findings in the live view of the sample or in the recorded images. We extracted 53 image features, including automated object detection (with a LoG filter) and texture features based on the grey‐level co‐occurrence matrix of the images, to characterize the spectrum of samples we investigated in this pilot study. Our random forest classifier trained on these features resulted in 95% accuracy, comparable to gold standard diagnostics (Figure 4 and Figure S14, Supporting Information). Beyond the binary classification of infection status, these rich image data could support further applications such as the detection of specific cell types or species, and other cellular phenotypes (e.g., sickle cell disease, other parasites such as Loa Loa), or drug resistance profiling, with further development of the image classification algorithms.^[^ ^60^, ^61^ ^]^ Insights from the feature importance (as in Figure 4B) can support new findings and further development of this method.

This platform's impact depends on its usability in resource‐constrained settings, as is common in malaria‐endemic areas. This platform is designed to be operable outside laboratory settings and to meet the WHO “ASSURED (Affordable, Sensitive, Specific, User‐friendly, Rapid and robust, Equipment‐free and Deliverable to end‐users)” criteria for effective diagnostic tests in resource‐limited settings.^[^ ^9^, ^11^ ^]^ The costs (breakdown in Figure S1B, Supporting Information) include materials for the device (100–1000× lower than the cost of a standard light microscope) and the per‐test cost (comparable to a single RDT test). Our test is highly accurate, with sensitivity and specificity on par with the performance of existing field diagnostics.^[^ ^33^ ^]^ Our approach minimizes the user‐intensive preparation steps so that this test can be performed without specific technical expertise, as highlighted by the positive feedback on device operation received from early prototype users. Device assembly is straightforward, and the design is modular and adjustable for different applications as needed. Since our testing involves minimal preparation, the sample‐to‐result can be accomplished in under 10 min. Sample processing can be multiplexed, with up to ten samples lysed and suspended simultaneously in separate levitation units, then inserted into the platform to be imaged serially. The levitation device itself is compact (5 × 1 × 1 cm) and portable (<100 g), reusable, and is interfaced with a cellphone attachment prototype for microscope‐free imaging and data connectivity even in locations without electricity. Further, it is amenable for smartphone‐based on‐demand global satellite positioning sample geo‐tagging for infection surveillance and control purposes. The platform is compatible with different smartphone models by replacing the removable phone holder insert. All reagents and components used for detection are ambient temperature‐stable, fast‐acting, and inexpensive at the volumes used per test. Without needing any laboratory infrastructure (e.g., refrigerator, incubator, and centrifuge) or electricity access (using batteries), this platform is both equipment‐free and deliverable to the end‐users in non‐laboratory settings. The platform thus has strong potential to aid surveillance and testing needs in relevant malaria‐endemic regions.

This prototype requires further development and testing to be implementable at broader scales, and to limit the need for individual troubleshooting. Saponin (being of natural origin) may have variable lysis efficacy, and batches from different sources should be pre‐tested for efficacy before use in clinical testing. While there is positive feedback for image capture on smartphones with increasing adoption, this may not be optimal for every setting, and an alternative design with inexpensive built‐in optics and sensors should be explored. Potential limitations of image‐based detection include error modalities in automated parasite detection such as 1) debris and artifacts, 2) edge cases in border regions, 3) out of focus particles, 4) clusters of cells, 5) changing parasite morphology due to motility, and 6) false‐negative cases (missing cells), potentially from unusual morphology. Going forward, performance can be improved to address many of these by training our model on a larger dataset and incorporating expert‐labeled image features in our detection approach. Another constraint of this study was the limited access to whole blood samples from malaria patients with low parasitemia since most samples collected were at least 1% parasitemia, due to the inclusion criteria for the ongoing study that sourced the samples. We estimated the performance of this technique at low parasitemia levels by simulating such samples with *P. falciparum* cultures spiked in whole blood, as seen in Figure 2B. Although early results are promising, this approach would further benefit from continued validation with a more extensive dataset (including lower‐parasitemia samples) to train the classifier (and explore deep learning network approaches). More training data would improve the robustness of the platform since clinical samples can be highly variable and may have co‐infections or co‐morbidities. Since the classifier is trained on *P. falciparum* patient samples, it is inherently tuned to detect ring‐stage parasite morphology since these are the predominant stage in circulation, with later stages (trophozoites, schizonts) sequestered in capillaries. Training on a larger and more varied dataset could enable stage‐specific detection capability, including non‐ring stages, for other applications such as *Plasmodium vivax* detection where later stages are found in circulation.^[^ ^62^ ^]^ Further, the use of a mobile camera or alternative optical sensor with greater resolution would enable stronger training data, for classification with better sensitivity and specificity to clinical samples as well as other quantifiable parameters of human blood samples and potential infection. Such steps can bring this platform closer to the WHO's ASSURED criteria and overcome logistical hurdles toward application in clinics (and beyond).

The demonstrated system combines mobile lysis, levitation, imaging, and automated computer vision‐assisted classification. It provides the basis for an innovative, accessible platform putting quantitative malaria sample analysis within reach of healthcare workers in resource‐limited settings. Beyond sensitive and specific detection of malaria, this platform could enable field applications of gametocyte isolation for transmission tracking, downstream analysis, and real‐time drug susceptibility profiling for emerging drug resistance threats. It can fit into the existing workflows at the nodes of diagnosis to provide additional capabilities with minimal auxiliary equipment and training, all with the benefits of comparable per‐use cost and ease of use of current RDTs. As infectious diseases strain healthcare systems globally and disproportionately devastate resource‐challenged regions, such flexible and low‐footprint analytical tools need to be both highly capable and broadly accessible. This platform can support healthcare workers tackling malaria detection challenges at the patient interface despite infrastructural constraints.

## 4 Experimental Section

### Levitation Suspension of Blood Cells in a Microscale Environment

The magnetic levitation platform, as pictured in Figure 1B,C, was a compact device that levitates cells in a prepared solution, shown to be capable of separating various cell types such as cancer cells from blood cells, including cultured ring‐stage *P. falciparum*‐infected RBCs from uninfected RBCs as shown in a previous work.^[^ ^42^, ^63^ ^]^ The solution was prepared with saponin (for lysis) and non‐cytotoxic chelated gadolinium ions (for levitation) in phosphate buffered saline (PBS) solution. The cells were suspended in this solution as in Figure 1B, with further reagents added per experimental needs, within a square channel in the magnetic field created between two permanent magnets. As shown in the steps throughout Figure 1B, cells and their fragments levitated over time as a function of their density and magnetic susceptibility with respect to the surrounding medium. At equilibrium, this levitation pattern was imaged and analyzed, using the platform in Figure 1C, producing images such as the examples in Figure 1D, which were used to extract insights into the cell population's biophysical characteristics with the workflow in Figure 1E.

### Assembly of Magnetic Levitation Chamber

The primary levitation device was assembled with two neodymium permanent bar magnets (5 cm × 0.5 cm × 0.2 cm) of 0.30 T each, with like poles facing each other and a gap of 1 mm between them, as shown in Figure 1B. These magnets were held in this configuration with two pieces of laser‐cut plastic. A square glass capillary (1 mm × 1 mm × 5 cm) containing the levitation mixture of cells was inserted into the gap between the magnets, and the device was placed in the cellphone‐interfaced portable imaging platform, described below.

### Cellphone‐Based Portable Modular Platform for Imaging Cell Suspensions

As depicted in Figure 1C, the portable imaging platform was assembled from several in‐house, customizable, 3D‐printed, and laser‐cut components made of polymethylmethacrylate. They were designed to fit the magnetic levitation chamber, its magnets, an Android smartphone, and additional optical components for illumination, magnification, and aberration correction. Once 3D‐printed from the STL files, the individual parts simply slotted together and were placed on a tabletop. The LED was soldered to a coin battery holder and placed in the platform. The system was designed to allow easy sample exchange, manual focus adjustment, and easy modulation of imaging parameters. The system components are listed in Note S1, Supporting Information, visually described in Figure S1A, Supporting Information, and the materials costs are shown in Figure S1B, Supporting Information. The cellphone was recharged using a high‐capacity (25Ah) lithium battery pack (ADDTOP HI‐S025 model) that contained solar panels, which was ideal for environments with infrequent electricity access (approximately once a month) or regular sunlight exposure, since either could be used to recharge the battery pack itself if the other was unavailable.

### Experiments with Cultured *Plasmodium falciparum* in Erythrocytes, and Uninfected Controls

The standard laboratory‐adapted strain of *P. falciparum*, 3D7, was cultured in human peripheral blood erythrocytes at 2% hematocrit, in complete RPMI medium with 0.5% albumax, at 37 °C in 5% carbon dioxide and 3% oxygen. Ring‐stage parasites (within 18 h of bursting) were synchronized by incubation in 5% sorbitol for 10 min at 37 °C. Parasitemia was calculated by counting Field's‐stained blood smears using a Miller reticle. Uninfected controls were prepared with de‐identified whole blood donor samples from the Stanford Blood Center. Preliminary experiments in laboratory settings, prior to field testing, were imaged on a standard microscope with a vertical light path, using Zeiss ZEN, Blue edition, software to collect microscope image data captured on an attached camera. Acridine orange staining (at 0.02% dilution, using Sigma Aldrich A9231‐10ML) was used to mark nucleic acid content (i.e., parasitic bodies or leukocytes, since erythrocytes themselves lack nuclei). The cell mixture was incubated with an acridine orange solution prepared in PBS at a ratio of 9:1 (cell mixture:acridine orange stock) for a minimum of 3 min at room temperature. In a previous work, acridine orange incubation was found to have no significant effect on the levitation height of RBCs or nucleated leukocytes.^[^ ^42^ ^]^ Fluorescence imaging was used with acridine orange‐stained samples on the microscope, with the DNA‐bound excitation peak at 502 nm and the emission peak at 525 nm.

### Lysis of Uninfected and *Plasmodium falciparum*‐Infected Erythrocytes via a Range of Lysis Agents

*Streptolysin O*: SLO was a membrane pore‐forming toxin from *Streptococcus pyogenes* that targeted cholesterol in cell membranes.^[^ ^64^ ^]^ Based on prior work that took advantage of this mechanism to induce selective lysis within a heterogeneous population of erythrocytes,^[^ ^44^ ^]^ an SLO‐based method was levied to specifically lyse uninfected erythrocytes (which had normal cholesterol levels by default) and leave the infected erythrocytes intact (which had depleted cholesterol due to parasitic activity), as described in Figure 2A[i]. SLO, from Sigma‐Aldrich (S5265‐25KU), was obtained as 25 000 units of lyophilized powder, reconstituted in 1 mL of PBS, and stored frozen in 40 µL aliquots. SLO was activated by mixing each of these aliquots with 144 µL of PBS with 0.1% fetal bovine serum albumin (from EMD Millipore, Cat. No. TMS‐013‐B) and 16 µL of 0.1 m DL‐dithiothreitol solution (Sigma Aldrich 646563‐10X.5ML). The mixture was then incubated at 37 °C for 2 h and could be stored temporarily (<1 month) at 4 °C. SLO treatment was performed on 3D7 cultured parasites reconstituted to the typical hematocrit of whole blood (≈50%). 2 µL of this blood was mixed with 2.8 µL of activated SLO solution and incubated at room temperature for 6 min for lysis to occur. This mixture was diluted to a final volume of 100 µL in the isotonic paramagnetic levitation solution in PBS.

While this form of lysis could be specific when precisely tuned, and the lysis occurred rapidly, it requires a time‐consuming preparation protocol (2+ h) and a relatively unstable product that depends on refrigeration, incubation, and thus electricity and external equipment. It also requires specialized training and higher cost. Thus, while SLO could induce selective lysis, it was also more dependent on external equipment and prone to inconsistency.

*Saponin*: Saponin was another biologically derived detergent used to lyse erythrocytes in malaria studies.^[^ ^46^, ^65^, ^66^ ^]^ While it leaves the intracellular parasite intact, it frees >90% of ring‐stage parasites to be released from erythrocytes in saponin‐induced hemolysis, as described in Figure 2A[ii].^[^ ^67^, ^68^ ^]^ Although less specific in erythrocyte lysis than SLO, saponin was rapid (<2 min), inexpensive, and widely available. Further, it did not need centrifugation for use, or refrigeration for storage in powdered form (or anecdotally in prepared solution for temporary storage, e.g., up to 2 months). One limitation of using saponin was the variability in saponin types available due to its natural origin, which calls for quality control testing if this protocol were to be consistently implemented at scale.

Dry saponin (Sigma‐Aldrich, 47036‐50G‐F) was reconstituted in PBS and sterilized by passing through a 0.2 µm filter. A stock solution was prepared to a concentration of 1% w/v, from which dilutions were prepared in PBS to maintain tonicity, as necessary. Similar to the SLO lysis protocol, a mixture of cultured parasites or whole blood was mixed with an isotonic saponin solution and incubated at room temperature, then observed for lysis. Lysis generally occurred instantly or within 5 min at the lowest concentrations tested. Saponin final concentrations from 0.05% w/v to 0.125% w/v were tested to visually determine the degree of lysis (observation of color changes with hemoglobin release and morphological examination of erythrocytes and leukocytes). A final saponin concentration of 0.07% w/v was found to be optimal for lysing erythrocytes and releasing the intracellular parasites without lysing the parasite plasma membrane. It was observed that sterilized saponin solutions of 1% w/v and below could be temporarily stored at room temperature (up to 2 months) without adverse effect on its lytic capacity.

*Tween‐20*: Tween‐20 (Sigma‐Aldrich, P1379) was a more consistently produced detergent that used a similar mechanism to lyse RBCs but was significantly slower (requiring overnight treatment), which rendered it ineffective for processing samples within a relevant timeframe of a rapid test (Figure 2A[iii]). Tween 20 was diluted in PBS and incubated with the same cell mixtures to test lysis effectiveness. Concentrations from 0.5% to 5% were tested, and cell mixtures were incubated for up to 12 h to observe lysis. At lower concentrations, little or no lysis occurred even at these extended incubation periods. At higher concentrations, lysis occurred but only after prolonged incubation periods and not within minutes. Thus, while it was inexpensive, commonly available, and straightforward to use, it was not rapid enough to meet the WHO's “ASSURED” criteria.^[^ ^11^ ^]^

To assess whether saponin‐lysed parasites could be visually detected and quantified in levitation suspensions, saponin lysis protocol was performed on serial dilutions of 3D7 *P. falciparum* cultures (RBCs only) stained with acridine orange to highlight parasite bodies. The suspensions were imaged in levitation chambers and the number of visually detected parasites in the fluorescent channels were quantified (with the size and morphology criteria set in the Laplacian of Gaussian algorithm, as further described below), and found good correlation (*R* ^2^ = 0.856) between expected parasitemia and detected parasitemia.

### Quantification of Saponin‐Lysed Parasites in Levitation Images

Experiments such as those in Figure 2B were conducted with leukocyte‐depleted blood samples, either containing parasites (cultured with 3D7 strain of *P. falciparum*) or blank (uninfected), and exposed to saponin as per the lysis protocol described above. Levitation suspensions were imaged on the standard laboratory microscope and processed using custom‐written code implemented in Python's Jupyter notebooks to detect parasite‐like objects using a LoG algorithm (later adapted for whole blood samples collected from the field). The code is described in detail in the Note S2, Supporting Information.

### Preparation of Lysed Parasites, Whole Blood, and Spiked Samples

Experiments such as those in Figure 2C were conducted with whole blood samples from de‐identified human donors (purchased from Stanford Blood Center) and cultured 3D7 parasites (sorbitol synchronized to ring stage), lysed according to the saponin‐based protocol described above. Images taken on the standard laboratory microscope were processed using custom‐written code implemented in Python Jupyter notebooks to extract the levitation height distributions of cells from binarized images, applying a Savitzky–Golay filter to smooth the distribution and detect peaks that represent lysed parasite bands and leukocyte bands.^[^ ^69^ ^]^ This code is described in detail in the Note S3, Supporting Information.

### Preparation of Whole Blood Samples from Malaria‐Infected Human Donors (*Plasmodium falciparum*) or Uninfected Human Donors, and Gold Standard Test Confirmation of Sample Infection Status

Experiments were conducted on small aliquots of de‐identified whole venous blood samples drawn in heparinized tubes for ongoing clinical work at Tororo District Hospital, Masafu General Hospital in Busia, and Nagongera Health Clinic IV, all in eastern Uganda, generously shared by researchers at the Infectious Diseases Research Collaboration of Uganda (IDRC‐Uganda). Samples were collected throughout July and August of 2019 during portions of both the local dry and wet seasons. The infection status of these samples was independently determined either by 1) Giemsa‐staining and counting of parasites on thick and thin blood smears by trained experts, or by 2) quantitative PCR. Samples were typically processed on the day they were drawn, or within 24 h with overnight refrigeration, which local researchers observed to be suitable for temporary sample preservation.

### Final Sample Preparation Protocol with Whole Blood Clinical Samples

The fresh whole blood aliquots described above were prepared as follows: 10 µL of whole blood was mixed with 5 µL of 1 mm gadobutrol (paramagnetic ions) and 35 µL of 0.1% w/v saponin solution in PBS using a micropipette. The mixture was transferred into the glass capillary and sealed on both ends with Critoseal, then inserted between the two magnets in the levitation device. Mixing and insertion typically took less than 1 min when buffers were prepared in advance. After 3 min, when both lysis and levitation equilibrium occurred, the sample was ready to image. The device was prepared for imaging by 1) inserting the smartphone into the holder and opening the camera app, 2) switching on the LED, and 3) sliding the capillary into the focal point. When the sample was ready, the cell lysate suspension was imaged on the smartphone camera.

The analysis was subsequently performed on a local or wirelessly connected computer by transporting the captured images using a USB cable, Bluetooth, Wifi, or near‐field communication.

### Laplacian‐Of‐Gaussian Algorithm for Detecting Parasite‐Like Objects in Images

Parasite‐like objects were detected in the whole blood‐lysed sample images taken on the portable imaging platform, using a LoG function. Once captured, the image could be passed to a custom Python function that uses modules including NumPy, OpenCV, and skimage to compute the number of parasite‐like objects and their locations, running in less than 10 s per image on a standard laptop computer. The final output was a key feature used in the classification of the sample images. The step‐by‐step function is described in Note S4, Supporting Information. The result was an output containing the locations of all detected objects that met the stipulated conditions. These could be overlaid on an image to check correlation, as in Figure 4A. The number of detected objects could be counted by the array shape, and was used as an indirect measure of sample parasitemia. The algorithm parameters were selected for maximum classification accuracy after testing a range of the parameters used above, as in Figure S6, Supporting Information.

### Gray‐Level Co‐Occurrence Matrix Analysis of Texture in Images

The mahotas module in Python was used, along with NumPy and OpenCV, to compute the GLCM for each image, and accordingly, 13 specific texture features (computed with 26 neighbors, and each along four directional axes: horizontal, vertical, left diagonal, and right diagonal).^[^ ^70^ ^]^

Pre‐processing steps before extracting the Haralick features using mahotas:

- Read the image file from its stored location.
- Convert from RGB (red‐green‐blue) to grayscale.
- Crop borders from image to standard dimensions.
- Invert image.
- Apply Gaussian blur with a kernel size of (11,11).

The output for each image was a total of 52 values; four values (for different directions) for each of the 13 features below:

1. Angular Second Moment, or Energy
2. Contrast
3. Correlation
4. Sum of Squares: Variance
5. Inverse Difference Moment
6. Sum Average
7. Sum Variance
8. Sum Entropy
9. Entropy
10. Difference Variance
11. Difference Entropy
12. First Informational Measure of Correlation
13. Second Informational Measure of Correlation

The directional means of each of these can be seen in Figures S7–S9, Supporting Information.

### Machine Learning with Extracted Features

Several classical machine learning algorithms were applied to the dataset of 53 morphological features extracted from the images (52 GLCM‐based texture features, with five examples in Figure 3D and directional means of all 13 in Figures S9–S11, Supporting Information, as well as the number of parasite‐like objects detected by LoG in Figure 3B) to assess the potential for improved classification by feature combination. The models were trained on a subset of the dataset, with known labels of malaria‐positive versus malaria‐negative. The full dataset contained 81 samples, each whole blood sample drawn from a unique human volunteer. Testing was conducted using cross‐validation with nine folds to estimate accuracy. The results of this preliminary assessment, with default hyperparameters, can be seen in Figure S11, Supporting Information.

After proceeding with the Random Forest model, GridSearchCV was used to tune the hyperparameters on the 75% training split of the dataset. The parameter space searched for “RandomForestClassifier” on scikit‐learn is described in Note S5, Supporting Information. Based on the results, the final random forest classifier applied was generated as shown in Figure 4A and Figure S12, Supporting Information, in scikit‐learn with the following parameters:

- Criterion: “gini” (similar performance, computationally efficient).
- Bootstrap used: true (to improve accuracy without overfitting).
- Maximum number of leaf nodes: 17 (to prevent overfitting).
- Minimum number of samples at each leaf node: 2.
- Minimum number of samples needed to split an internal node: 2.
- Number of estimators: 10 (to retain computational efficiency after fitting was achieved).

Exported .viz files could be used to visualize decision trees such as those in Figure 4A and Figure S12, Supporting Information. Graphviz files could be processed on any relevant applications of choice; a web application was used as noted here: http://www.webgraphviz.com/.

### Statistical Analysis

The z‐test was used to compare between means of two groups with known variances and sample size above 50 (as in Figure 3B,D and Figures S7–S9, Supporting Information). Otherwise, Welch's *t*‐test was used to compare between two means (as in Figure 3C). Test statistics, sample size, and *p*‐values were reported in each case where the data were presented.

In Figure 4C,D and Figure S14, Supporting Information, the confusion matrices reported the number of samples in each group that were classified by the gold‐standard method, “actual” (expert microscopy or qPCR), and by the new method, “predicted” (levitation image features in random forest classifier), as malaria‐positive or malaria‐negative. These numbers could be broken down as follows and used to quantify the accuracy of the classifier.

- TPV (true positive value): number of positive samples correctly classified as positive.
- FNV (false negative value): number of positive samples incorrectly classified as negative.
- FPV (false positive value): number of negative samples incorrectly classified as positive.
- TNV (true negative value): number of negative samples correctly classified as negative.
- Sensitivity, or TPR (true positive rate): TP/(TP + FN)
- Specificity, or TNR (true negative rate): TN/(TN + FP)
- Accuracy: (TP + TN)/(TP + FN + TN + FP).

Limit of detection and limit of blank was calculated as follows:^[^ ^47^ ^]^

- Limit of Blank (LoB) = mean of blank samples + 1.645 (S.D. of blank samples)
- Limit of Detection (LoD) = LoB + 1.645 (SD of low concentration samples)
- These were calculated for a standard deviation covering 95% of the Gaussian distribution.

### Human Subjects Statement

All experiments presented in this study were performed in compliance with the guidelines of the relevant institutions, under the approval of protocols approved by the University of California Institutional Review Board (IRB) (#16‐19084, #16‐20562, and #17‐22544) and the Stanford University IRB (Protocol #40464). Under these protocols, informed consent was obtained when required before involvement with human subjects. Any identifying information associated with the acquired samples was removed before being used within the scope of this study, so that only de‐identified samples were used, for the protection of the subjects' identity.

## Conflict of Interest

U.D. is a founder of and has equity interest in: 1) DxNow Inc., developing sperm sorting tools, 2) Koek Biotech, developing microfluidic tools, 3) Levitas Inc., developing microfluidic products for rare cells, 4) Hillel Inc., developing microfluidic cell phone testing, and 5) Mercury Biosciences, developing vesicle isolation tools. U.D.'s interests were viewed and managed in accordance with conflict‐of‐interest policies. The remaining authors declare no conflict of interest.

## Authors Contributions

Conceptualization: S.S.D., B.G., E.S.E., U.D.; Methodology: S.S.D., O.B., P.T., B.G., E.S.E., U.D.; Software: S.S.D.; Validation: S.S.D., O.B.; Formal analysis: S.S.D., D.A.; Investigation: S.S.D., O.B., P.T.; Resources: O.B., P.T., B.G., E.S.E., U.D.; Writing—original draft: S.S.D.; Writing—review and editing: S.S.D., O.B., P.T., D.A., E.S.E., U.D.; Visualization: S.S.D.; Supervision: B.G., E.S.E., U.D.; Project administration: P.T.; Funding acquisition: S.S.D., E.S.E., U.D.

## Supporting information

Supporting Information

## Data Availability

The data that support the findings of this study are available from the corresponding author upon reasonable request.
