# Supplementary material for: Automated Recognition of Plasmodium falciparum Parasites from Portable Blood Levitation Imaging
Source: Adv Sci (Weinh). 2022 Aug 11;9(28):2105396. doi: 10.1002/advs.202105396 (PMC9534981; doi:10.1002/advs.202105396)
Supplement: Supplementary file 1 — Supporting Information [file ADVS-9-2105396-s001.pdf]

ADVANCED SCIENCE
Open Access

Supporting Information

for *Adv. Sci.*, DOI 10.1002/advs.202105396

Automated Recognition of *Plasmodium falciparum* Parasites from Portable Blood Levitation Imaging

*Shreya S. Deshmukh, Oswald Byaruhanga, Patrick Tumwebaze, Demir Akin, Bryan Greenhouse, Elizabeth S. Egan and Utkan Demirci**

List of supplementary materials.

1. Note 1. Optical system components list.
2. Note 2. Laplacian-of-Gaussian algorithm for detecting parasite-like objects in microscope images.
3. Note 3. Image analysis algorithm to calculate levitation height distributions in Python.
4. Note 4. Laplacian-of-Gaussian algorithm for detecting parasite-like objects in cellphone images.
5. Note 5. Hyperparameter space searched for RandomForestClassifier on scikit-learn.
6. Figure S1. Assembly of portable imaging platform.
7. Figure S2. Optimizing parasite-leukocyte separation conditions with saponin lysis & levitation of parasite-spiked whole blood.
8. Figure S3. Example close-up images of ring-stage parasite morphology.
9. Figure S4. Image processing pipeline: two potential analysis pathways.
10. Figure S5. Example images of lysed whole blood containing parasites and leukocytes.
11. Figure S6. Optimizing parameters for blob detection via Laplacian of Gaussian operation.
12. Figure S7. Full quantification of all 13 Haralick's texture features from GLCM (1-5).
13. Figure S8. Full quantification of all 13 Haralick's texture features from GLCM (6-10).
14. Figure S9. Full quantification of all 13 Haralick's texture features from GLCM (11-13).
15. Figure S10. Principal Component Analysis of dataset.
16. Figure S11. Comparing various classical machine learning models for classification potential with blob and texture features.
17. Figure S12. Random Forest classifier estimator diagrams.
18. Figure S13. Relative importance of all features.
19. Figure S14. Confusion matrix of Random Forest Classifier applied to full dataset.
20. Figure S15. Levitation heights of leukocytes from whole blood.
21. Video 1. Example video capture of whole blood levitation suspension on portable platform, from initial timepoint to equilibrium (malaria-positive sample).
22. Video 2. Example video capture of whole blood levitation suspension on portable platform, from initial timepoint to equilibrium (malaria-negative sample).

Supplementary materials: Detailed Methods

Note 1: Optical system components list.

The main 3D-printed body holds (in order):

- Power source
    - coin battery
- Light source
    - 1W white LED
    - held in laser-cut PMMA holder
- Emission filter (optional)
    - for fluorescence imaging
- Sample holder
    - Laser-cut PMMA holder
    - holds the magnets and capillary in between
- Magnification
    - AmScope 20X objective lens
- Excitation filter (optional)
    - for fluorescence imaging
- Aspheric lens
    - Spherical aberration correction

This section slots into a secondary interlocking 3D-printed component as follows:

- 3D-printed removable phone holder

    - Custom for: Redmi Note 7 smartphone (can be customized to fit other smartphone's dimensions)
- Smartphone, with the camera facing the main body and screen facing the operator.

Note 2: Laplacian-of-Gaussian algorithm for detecting parasite-like objects in microscope images

Once captured, the image can be passed to a custom Python function that uses modules including numpy, OpenCV, and skimage to compute the number of parasite-like objects and their locations, running in less than 10 seconds per image on a standard laptop computer. The final output was evaluated as a proxy for quantifying parasitemia. The step-by-step function runs as follows:

- Read image file from its stored location.
- Convert from RGB (red-green-blue) to grayscale (i.e. 3-dimensional to 2-dimensional).
- Crop borders from image to standard dimensions:
- Detect midpoint of capillary by binarizing image to determine range of the capillary region in the image
- Cap image to 90% of image height around the detected midpoint of capillary, to reduce border artifacts that occur at high-contrast areas around capillary edges.
- Include condition to prevent capillary region being excessively cropped
- Apply Gaussian blur with kernel size of (5,5).
- Use “blob_log” function from skimage.feature:
- Minimum standard deviation for Gaussian kernel is set to 2.
- Maximum standard deviation for Gaussian kernel is set to 8.
- The number of intermediate values of standard deviations to consider in between is set to 6 (i.e. step size of 1).
- The threshold (absolute lower bound for scale space maxima) is set to 0.03.

- The result of the above step is an output containing the locations of all detected objects that meet the stipulated conditions. The number of such objects can be counted by the shape of the array and used as an indirect measure of the parasitemia of the sample.

Note 3: Image analysis algorithm to calculate levitation height distributions in Python

All images were imported, pre-processed, and analyzed using open-source Python 3.7 in the form of a Jupyter notebook for visual accessibility. The following modules were used: os for handling directories, NumPy for numerical computation and handling arrays, SciPy for scientific computation and statistical analysis, csv for spreadsheet import and export, matplotlib for plot generation, and cv2 and skimage for image processing. Further overview of image analysis and subsequent data analysis can be found in the Methods section. Following is the step-by-step workflow:

1. Import modules and functions.
2. Define file path to images.
3. Import image as RGB.
4. Convert image from RGB format (3-dimensional array) to grey format (2-dimensional array).
5. Rotate image to parallelize chamber boundaries with image boundaries in order to compensate for errors in microscope alignment.
6. Crop image to chamber boundaries.
7. Stretch or shrink image (while maintaining proportions) to compensate for different sized images from different microscopes.
8. Save cropped image for future analysis.
9. Apply Gaussian smoothing to reduce high-frequency noise.
10. Apply Laplacian filter to reduce low-frequency noise.

11. Binarize image using Otsu's thresholding so that it now represents cell-containing pixels and non-cell-containing pixels. (Use binary thresholding with threshold set to 5 if image contrast is too low).
12. Crop 0.5% image boundary to remove false cell detection at boundaries.
13. If imaging artifacts or debris persist through low-frequency noise removal; set the values of those rows to zero manually.
14. Sum (binary) intensity values across each row to acquire a heightwise list of the sum of cell-containing pixels.
15. Subtract average background noise.
16. Center the distribution according to image so that the height distribution accurately reflects the height of the chamber.
17. Plot the array that represents the height distribution of cell-containing pixels in each image.
18. Apply Savitzky-Golay filter (using the savgol_filter function) to the height distribution of cell-containing pixels, with a window size of 87 and polynomial order of 4.
19. Plot the smoothed distributions and mark the leukocyte and parasite peaks.

Note: The equivalent of the method described above is also performed for images with both brightfield and fluorescent channel counterparts. The cell-containing pixels from the fluorescent channel are subtracted from those in the brightfield image to estimate non-fluorescent cell locations and fluorescent cell locations without double counting.

Note 4: Laplacian-of-Gaussian algorithm for detecting parasite-like objects in cellphone images

The step-by-step function runs as follows:

1. Read the image file from its stored location.
2. Convert from RGB (red-green-blue) to grayscale (i.e. 3-dimensional to 2-dimensional).
3. Crop borders from image to standard dimensions.
4. Invert image.
5. Apply Gaussian blur with a kernel size of (11,11).
6. Use "blob_log" function from skimage.feature:
7. Minimum standard deviation for the Gaussian kernel is set to 1.
8. Maximum standard deviation for the Gaussian kernel is set to 6.
9. The number of intermediate values of standard deviations to consider in between is set to 5 (i.e., step size of 1).
10. The threshold (the absolute lower bound for scale space maxima) is set to 0.03.
11. The result of the above step is an output containing the locations of all detected objects that meet the stipulated conditions. These can be overlaid on an image to check correlation, as in **Fig. 4A**. The number of detected objects can be counted by the array shape, and is used as an indirect measure of sample parasitemia.

Note 5: Hyperparameter space searched for RandomForestClassifier on scikit-learn

- Node impurity measure, 'criterion': "gini", "entropy".
- Maximum number of terminal nodes, ‘max_leaf_nodes’: 2 to 20.
- Minimum number of data points per leaf node: ‘min_samples_leaf’: 1, 2, 4, 8.
- Minimum number of data points in a node before it is split, ‘min_samples_split’: 1, 2, 4, 8, 10, 20.
- Number of trees in the forest, ‘n_estimators’: 1, 2, 3, 4, 5, 6, 7, 8, 9, 10, 20, 30, 40, 50, 60, 70.

A

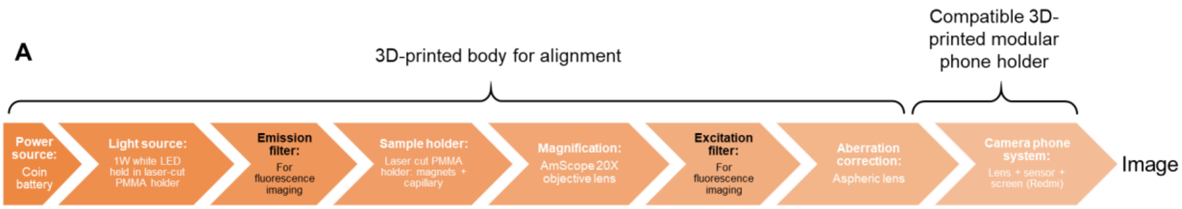

B

Materials	Cost (USD)
Platform assembly (reusable)<br>*Magnets, laser-cut holders, lenses, LED, etc.*	**$31.14**
Cellphone (Redmi 5)	~$180
Per-test materials<br>*Capillary, reagents*	**$1.16**
With FEP channels	$0.10

Figure S1. Assembly of portable imaging platform.

(A) Optical path in portable imaging prototype (white = main component; gray = optional component for additive capabilities).

(B) Estimated material cost of prototype (at small scales).

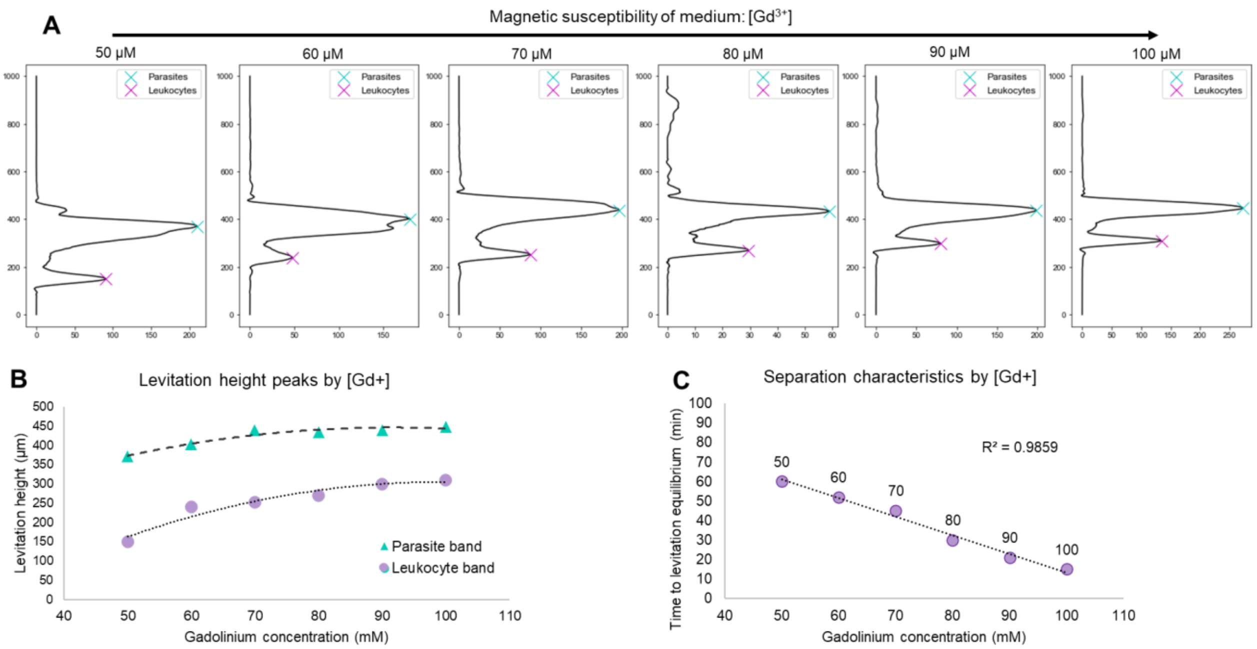

Figure S2. Optimizing parasite-leukocyte separation conditions with saponin lysis & levitation of parasite-spiked whole blood.

(A) Height distributions of parasite-spiked whole blood with saponin lysis, at varying medium magnetic susceptibilities.

(B) Quantification of separation in levitation height between the parasite peak and leukocyte peak, over medium magnetic susceptibility.
(C) Quantification of time to levitation height equilibrium, over medium magnetic susceptibility.

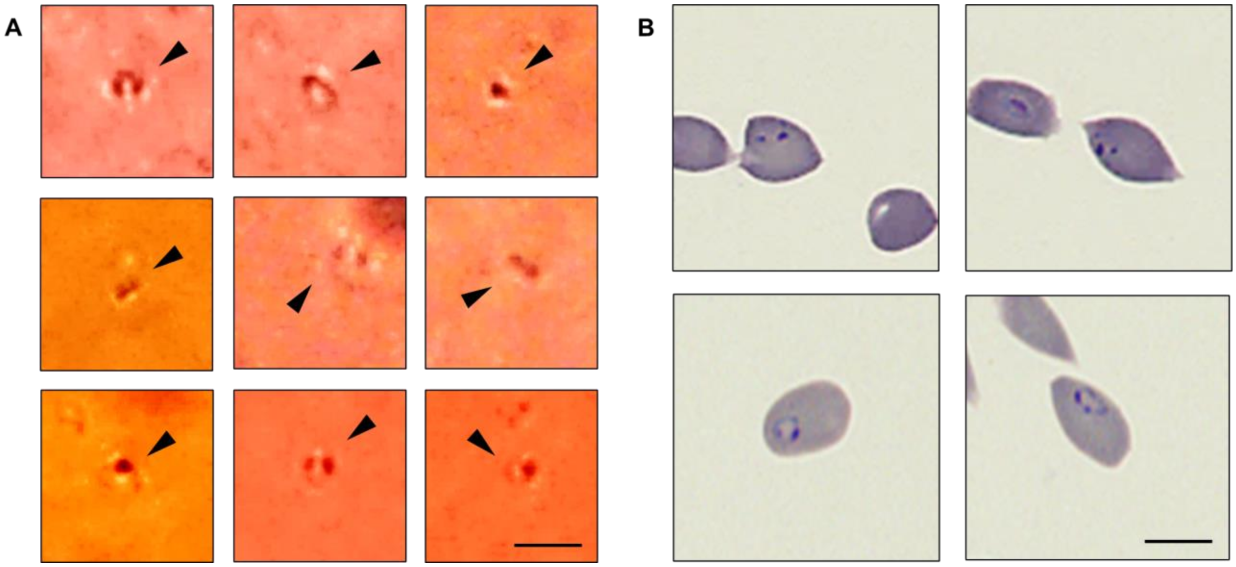

Figure S3. Example close-up images of ring-stage parasite morphology.

Example images in **(A)** ghosted RBC suspension (after saponin lysis) imaged on cellphone vs. **(B)** Field-stained fixed smears imaged on microscope. Scale bar is 5 μm in both.

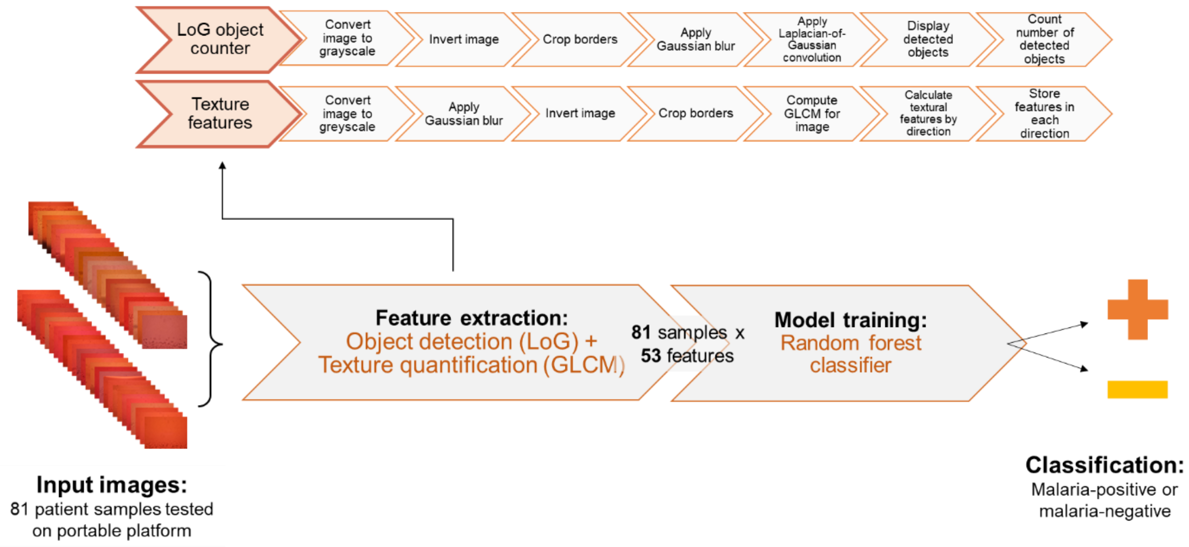

Figure S4. Image processing pipeline: feature extraction and classifier training.

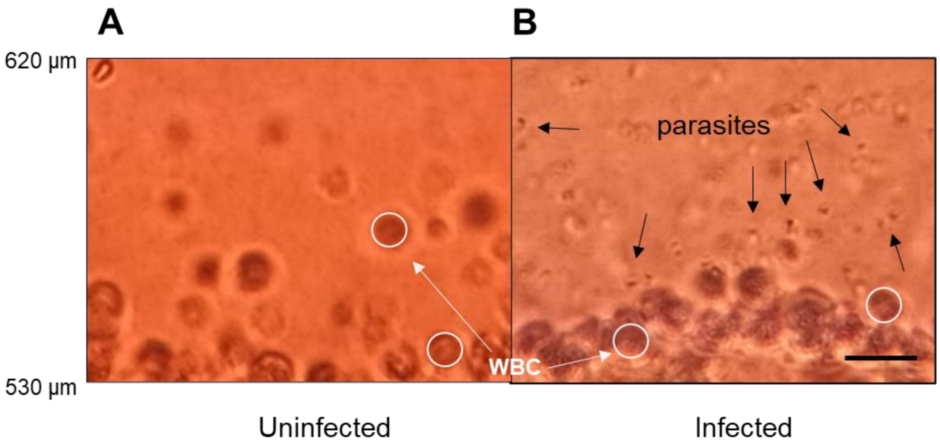

Figure S5. Example images of lysed whole blood containing parasites and leukocytes. **(A)** Uninfected example. **(B)** Infected example. Scale bar = 20 μm.

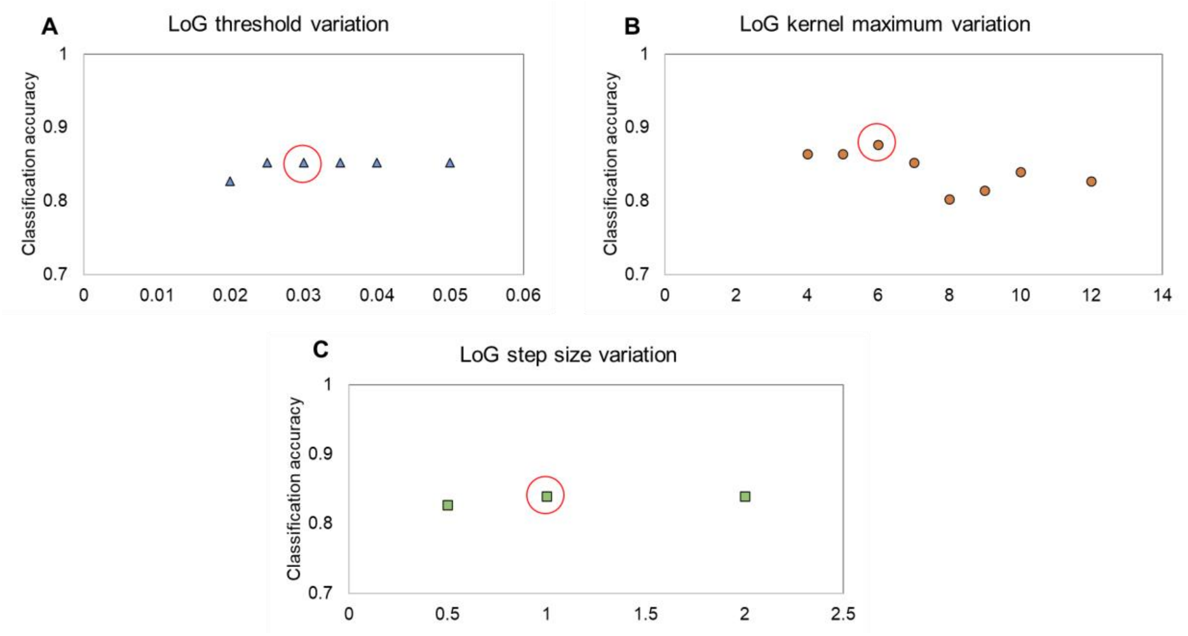

Figure S6. Optimising parameters for blob detection via Laplacian of Gaussian operation.

(A) Testing varying thresholds. **(B)** Testing varying kernel maximums. **(C)** Testing varying step sizes.

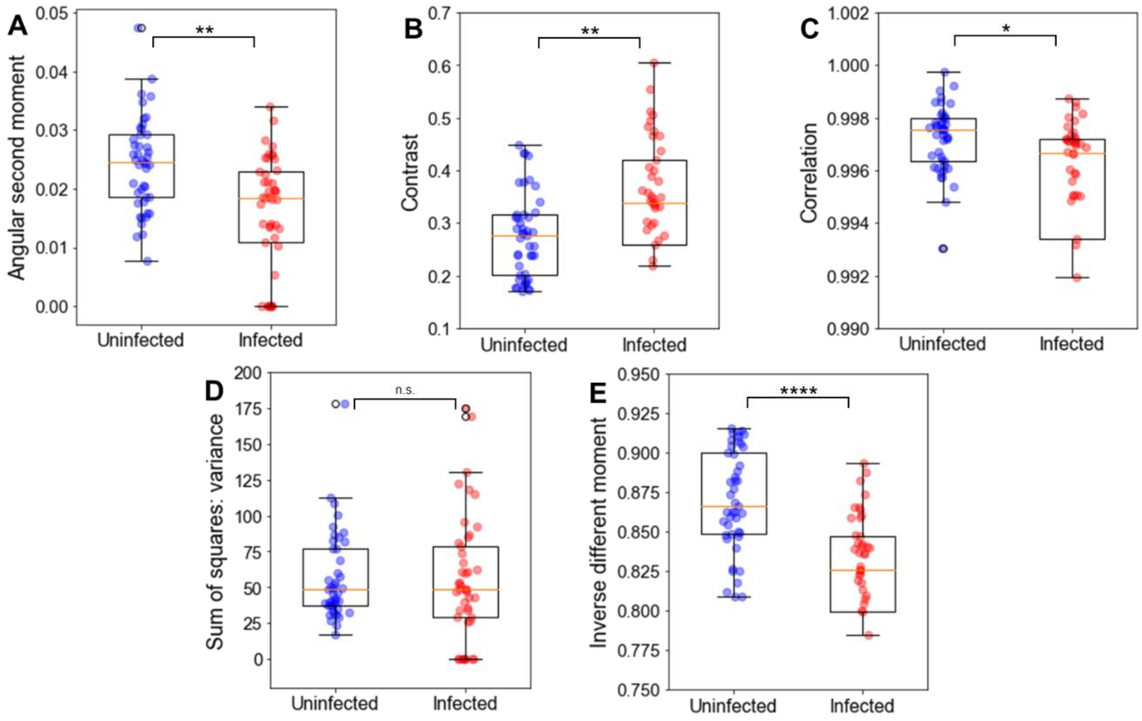

Figure S7. Full quantification of all 13 Haralick's texture features from GLCM (1-5).

The z-test was performed to compare uninfected and infected sample groups for each metric. The test statistic and p-value is reported for each.

(A) Angular second moment. Test statistic: 2.7. P-value: 0.0076.

(B) Contrast: Test statistic: -3.1. P-value: 0.0020.

(C) Correlation: Test statistic: 2.3. P-value: 0.023.

(D) Sum of squares variance: Test statistic: 0.23. P-value: 0.82.

(E) Inverse difference moment: Test statistic: 4.4. P-value: 0.000013.

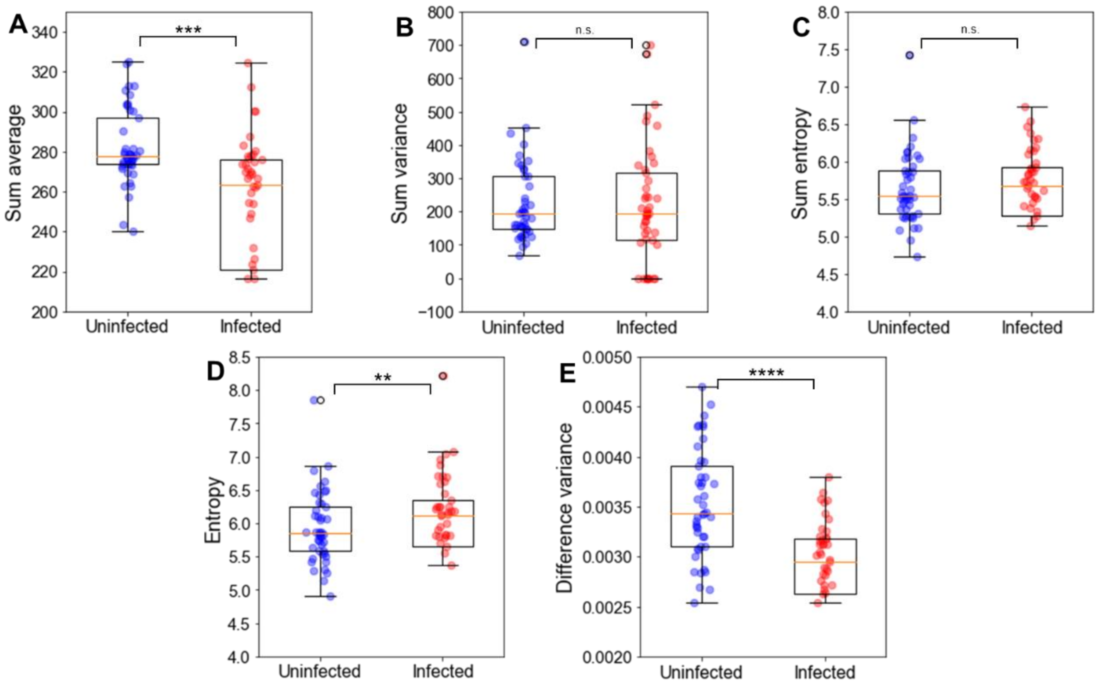

Figure S8. Full quantification of all 13 Haralick's texture features from GLCM (6-10).

The z-test was performed to compare uninfected and infected sample groups for each metric. The test statistic and p-value is reported for each.

(A) Sum average. Test statistic: 3.3. P-value: 0.00082.

(B) Sum variance: Test statistic: 0.23. P-value: 0.82.

(C) Sum entropy: Test statistic: -1.9. P-value: 0.064.

(D) Entropy: Test statistic: -2.7. P-value: 0.0068.

(E) Difference variance: Test statistic: 4.5. P-value: 0.0000057.

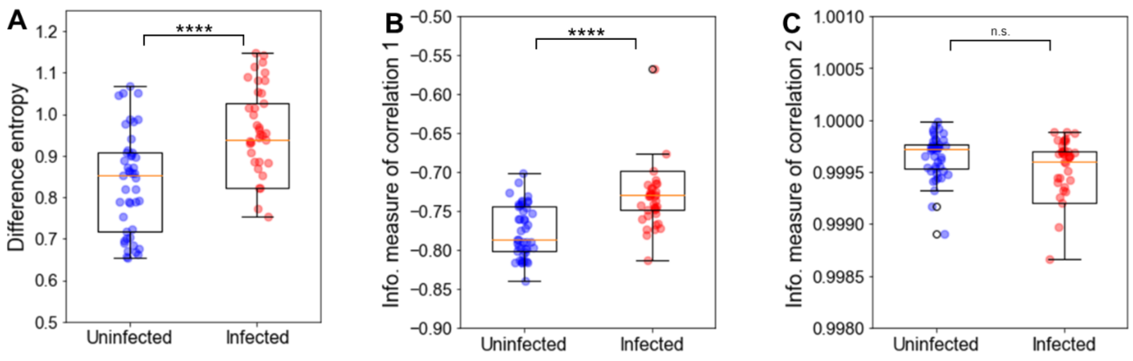

Figure S9. Full quantification of all 13 Haralick's texture features from GLCM (11-13).

The z-test was performed to compare uninfected and infected sample groups for each metric. The test statistic and p-value is reported for each.

(A) Difference entropy. Test statistic: -4.8. P-value: 0.0000020.

(B) Informational measure of correlation 1: Test statistic: -4.9. P-value: 0.00000076.

(C) Informational measure of correlation 2: Test statistic: 1.5. P-value: 0.15.

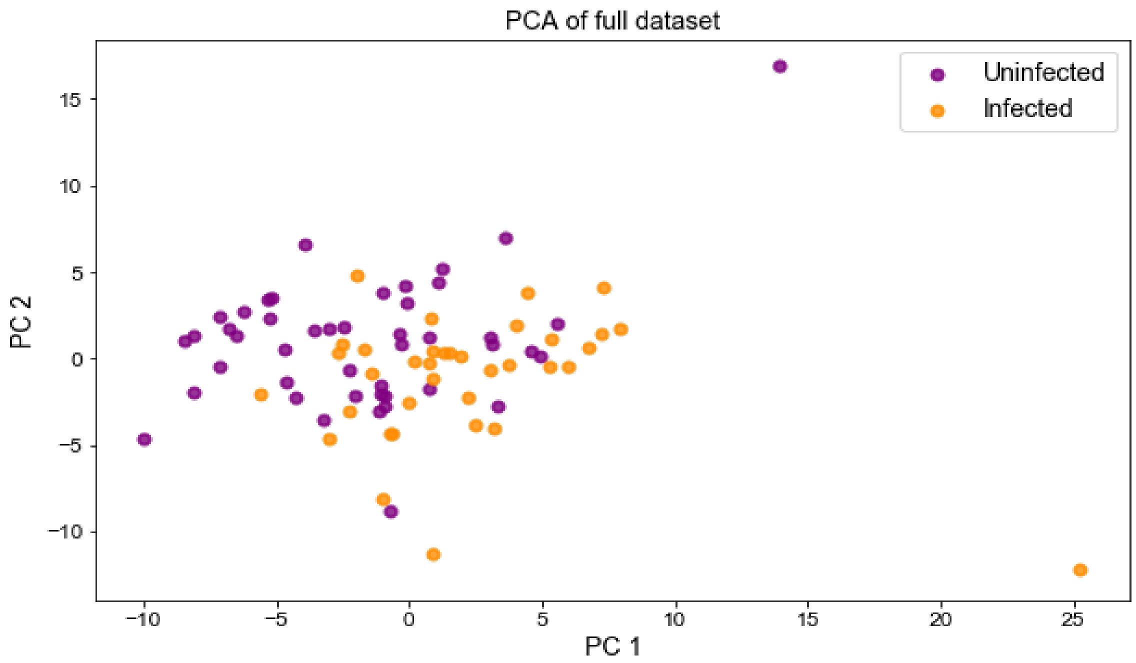

Figure S10. Principal Component Analysis of dataset

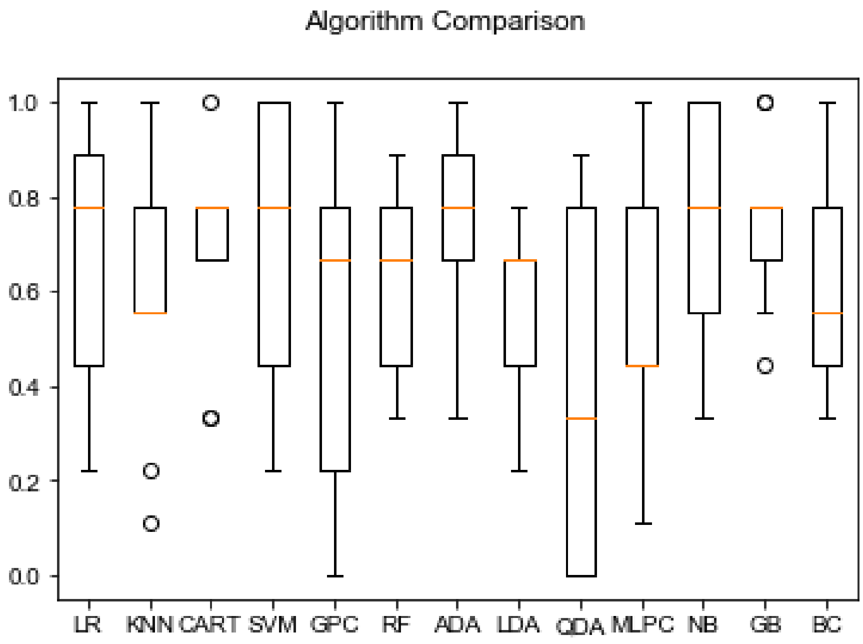

Figure S11. Comparing various classical machine learning models for classification potential with blob and texture features.

LR = Logistic Regression; KNN = K-Nearest Neighbors; CART = Decision Trees, SVM = Support Vector Classifier, GPC = Gaussian Process Classifier, RF = Random Forest Classifier, ADA = AdaBoost Classifier, LDA = Linear Discriminant Analysis, QDA = Quadratic Discriminant Analysis, MLPC = Multilayer Perceptron Classifier, NB = Gaussian Naïve Bayes, GB = Gradient Boosted Classifier, BC = Bagging Classifier.

Each element in the box-and-whisker plots represents the following: center line represents the median; box limits represent the upper and lower quartiles; whiskers represent the range of the data, circles represent outliers in the data.

Figure S12. Random Forest classifier estimator diagrams.

The following slides contain a diagram of each of the 10 estimators used in the Random Forest classifier (described in Fig. 4A).

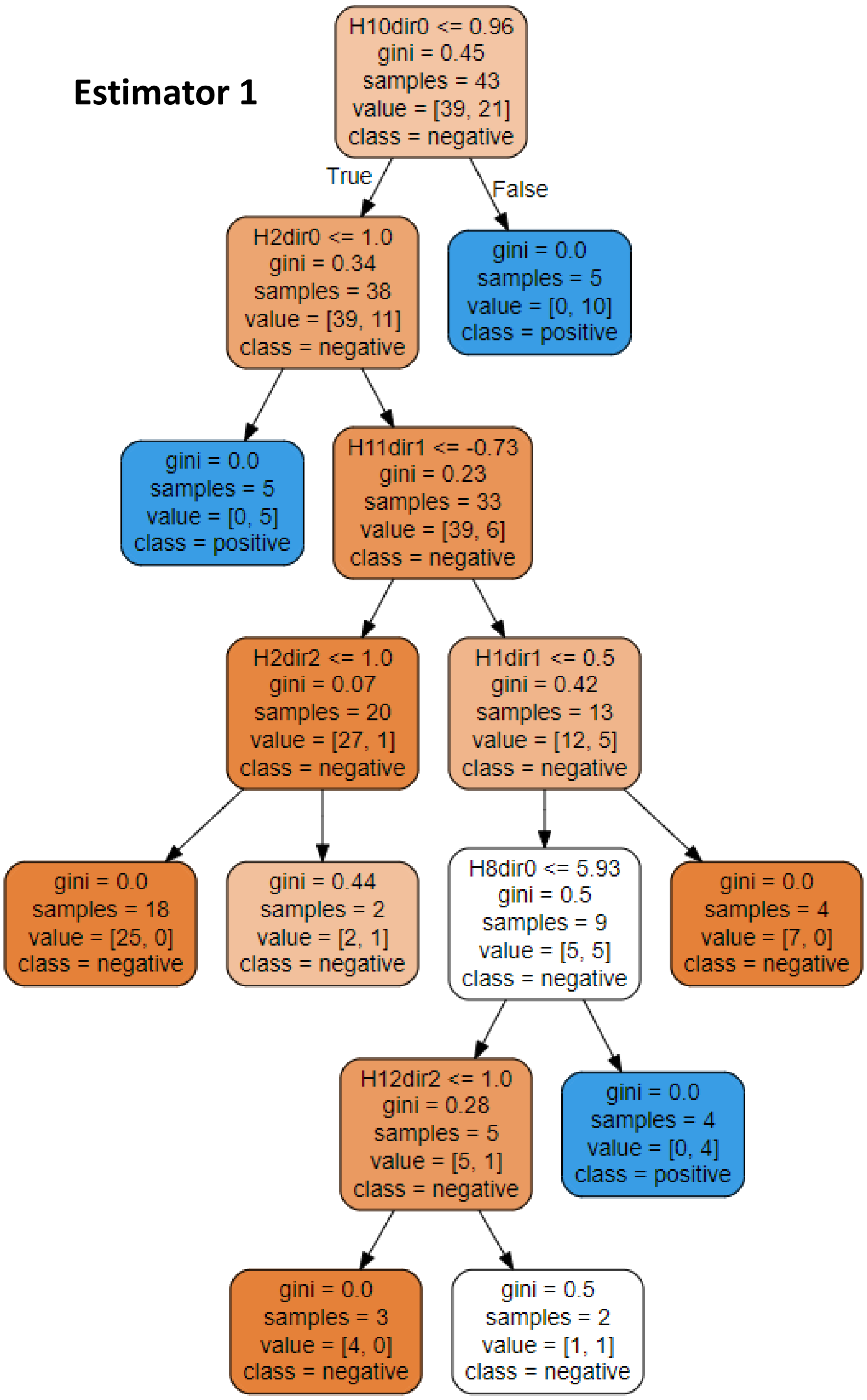

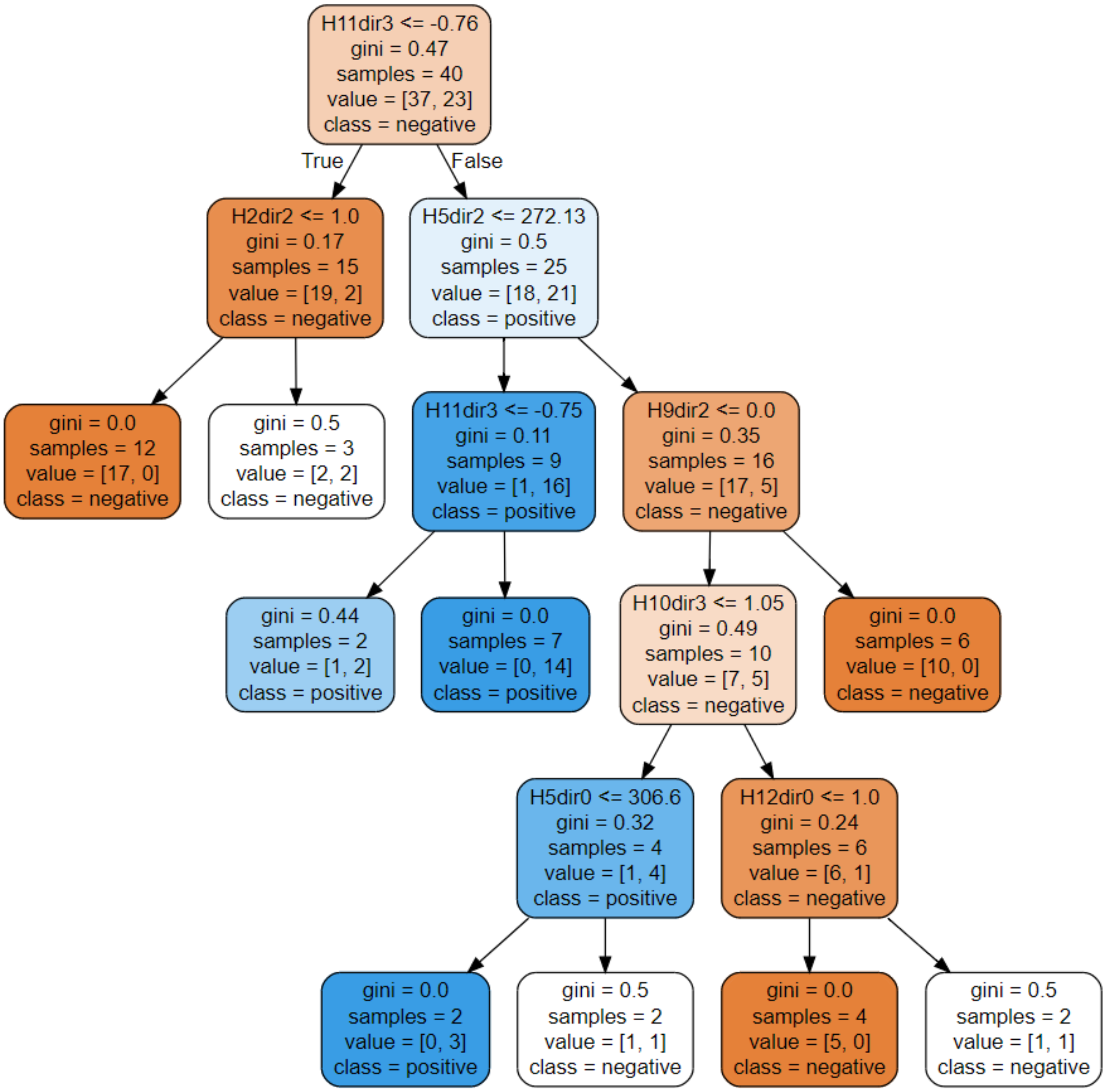
H11dir3 <= -0.76
gini = 0.47
samples = 40
value = [37, 23]
class = negative
True
False
H2dir2 <= 1.0
gini = 0.17
samples = 15
value = [19, 2]
class = negative
H5dir2 <= 272.13
gini = 0.5
samples = 25
value = [18, 21]
class = positive
gini = 0.0
samples = 12
value = [17, 0]
class = negative
gini = 0.5
samples = 3
value = [2, 2]
class = negative
H11dir3 <= -0.75
gini = 0.11
samples = 9
value = [1, 16]
class = positive
H9dir2 <= 0.0
gini = 0.35
samples = 16
value = [17, 5]
class = negative
gini = 0.44
samples = 2
value = [1, 2]
class = positive
gini = 0.0
samples = 7
value = [0, 14]
class = positive
H10dir3 <= 1.05
gini = 0.49
samples = 10
value = [7, 5]
class = negative
gini = 0.0
samples = 6
value = [10, 0]
class = negative
H5dir0 <= 306.6
gini = 0.32
samples = 4
value = [1, 4]
class = positive
H12dir0 <= 1.0
gini = 0.24
samples = 6
value = [6, 1]
class = negative
gini = 0.0
samples = 2
value = [0, 3]
class = positive
gini = 0.5
samples = 2
value = [1, 1]
class = negative
gini = 0.0
samples = 4
value = [5, 0]
class = negative
gini = 0.5
samples = 2
value = [1, 1]
class = negative

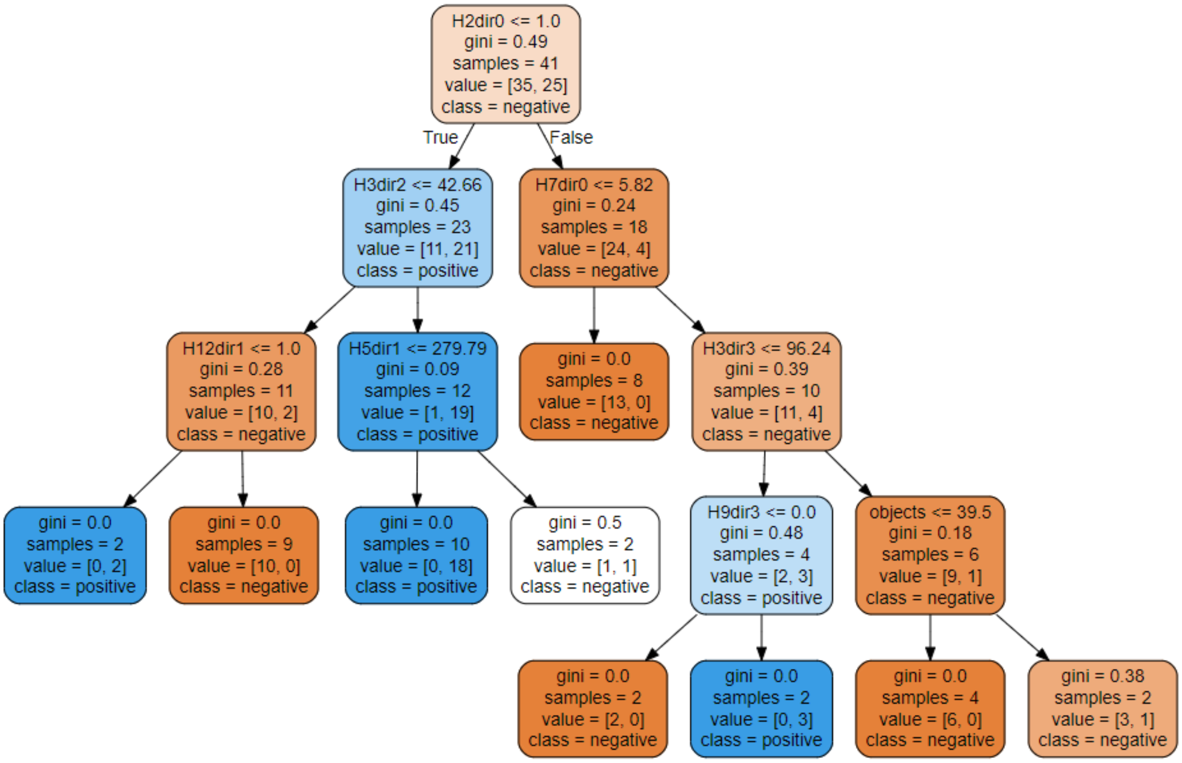
H2dir0 <= 1.0
gini = 0.49
samples = 41
value = [35, 25]
class = negative
True
False
H3dir2 <= 42.66
gini = 0.45
samples = 23
value = [11, 21]
class = positive
H7dir0 <= 5.82
gini = 0.24
samples = 18
value = [24, 4]
class = negative
H12dir1 <= 1.0
gini = 0.28
samples = 11
value = [10, 2]
class = negative
H5dir1 <= 279.79
gini = 0.09
samples = 12
value = [1, 19]
class = positive
gini = 0.0
samples = 8
value = [13, 0]
class = negative
H3dir3 <= 96.24
gini = 0.39
samples = 10
value = [11, 4]
class = negative
gini = 0.0
samples = 2
value = [0, 2]
class = positive
gini = 0.0
samples = 9
value = [10, 0]
class = negative
gini = 0.0
samples = 10
value = [0, 18]
class = positive
gini = 0.5
samples = 2
value = [1, 1]
class = negative
H9dir3 <= 0.0
gini = 0.48
samples = 4
value = [2, 3]
class = positive
objects <= 39.5
gini = 0.18
samples = 6
value = [9, 1]
class = negative
gini = 0.0
samples = 2
value = [2, 0]
class = negative
gini = 0.0
samples = 2
value = [0, 3]
class = positive
gini = 0.0
samples = 4
value = [6, 0]
class = negative
gini = 0.38
samples = 2
value = [3, 1]
class = negative

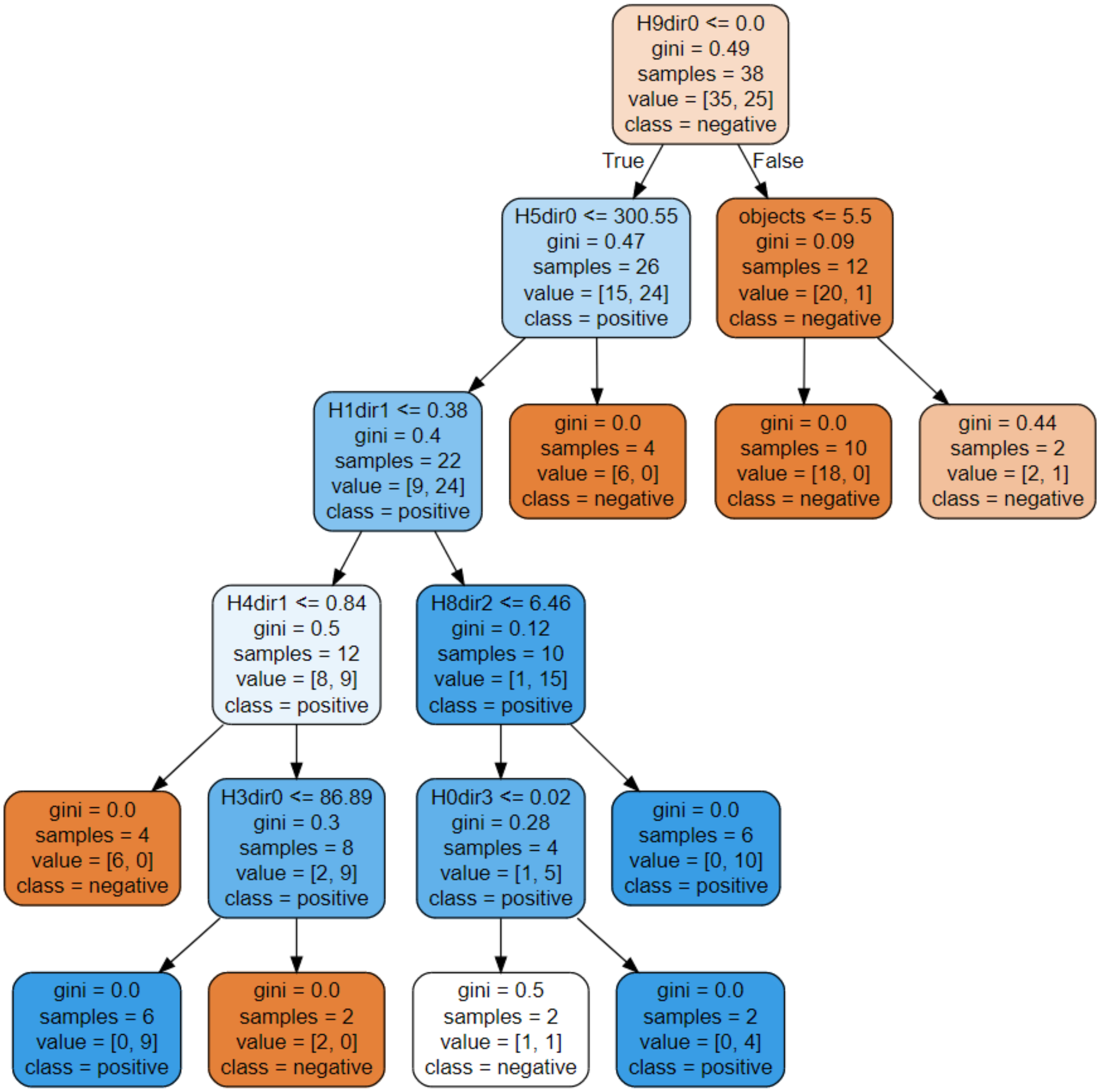
H9dir0 <= 0.0
gini = 0.49
samples = 38
value = [35, 25]
class = negative
True
False
H5dir0 <= 300.55
gini = 0.47
samples = 26
value = [15, 24]
class = positive
objects <= 5.5
gini = 0.09
samples = 12
value = [20, 1]
class = negative
H1dir1 <= 0.38
gini = 0.4
samples = 22
value = [9, 24]
class = positive
gini = 0.0
samples = 4
value = [6, 0]
class = negative
gini = 0.0
samples = 10
value = [18, 0]
class = negative
gini = 0.44
samples = 2
value = [2, 1]
class = negative
H4dir1 <= 0.84
gini = 0.5
samples = 12
value = [8, 9]
class = positive
H8dir2 <= 6.46
gini = 0.12
samples = 10
value = [1, 15]
class = positive
gini = 0.0
samples = 4
value = [6, 0]
class = negative
H3dir0 <= 86.89
gini = 0.3
samples = 8
value = [2, 9]
class = positive
H0dir3 <= 0.02
gini = 0.28
samples = 4
value = [1, 5]
class = positive
gini = 0.0
samples = 6
value = [0, 10]
class = positive
gini = 0.0
samples = 6
value = [0, 9]
class = positive
gini = 0.0
samples = 2
value = [2, 0]
class = negative
gini = 0.5
samples = 2
value = [1, 1]
class = negative
gini = 0.0
samples = 2
value = [0, 4]
class = positive

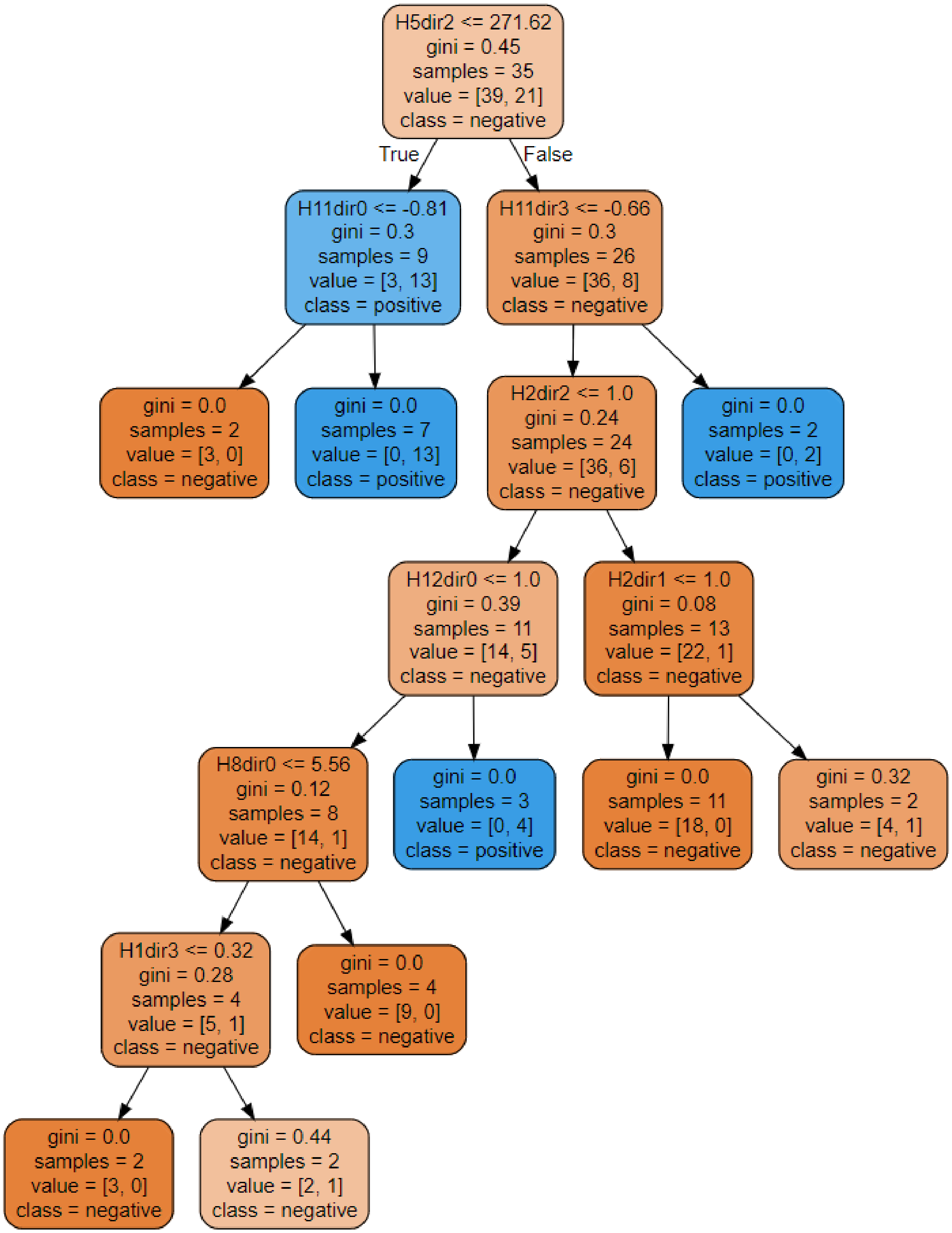

H5dir2 <= 271.62
gini = 0.45
samples = 35
value = [39, 21]
class = negative
True
False
H11dir0 <= -0.81
gini = 0.3
samples = 9
value = [3, 13]
class = positive
H11dir3 <= -0.66
gini = 0.3
samples = 26
value = [36, 8]
class = negative
gini = 0.0
samples = 2
value = [3, 0]
class = negative
gini = 0.0
samples = 7
value = [0, 13]
class = positive
H2dir2 <= 1.0
gini = 0.24
samples = 24
value = [36, 6]
class = negative
gini = 0.0
samples = 2
value = [0, 2]
class = positive
H12dir0 <= 1.0
gini = 0.39
samples = 11
value = [14, 5]
class = negative
H2dir1 <= 1.0
gini = 0.08
samples = 13
value = [22, 1]
class = negative
H8dir0 <= 5.56
gini = 0.12
samples = 8
value = [14, 1]
class = negative
gini = 0.0
samples = 3
value = [0, 4]
class = positive
gini = 0.0
samples = 11
value = [18, 0]
class = negative
gini = 0.32
samples = 2
value = [4, 1]
class = negative
H1dir3 <= 0.32
gini = 0.28
samples = 4
value = [5, 1]
class = negative
gini = 0.0
samples = 4
value = [9, 0]
class = negative
gini = 0.0
samples = 2
value = [3, 0]
class = negative
gini = 0.44
samples = 2
value = [2, 1]
class = negative

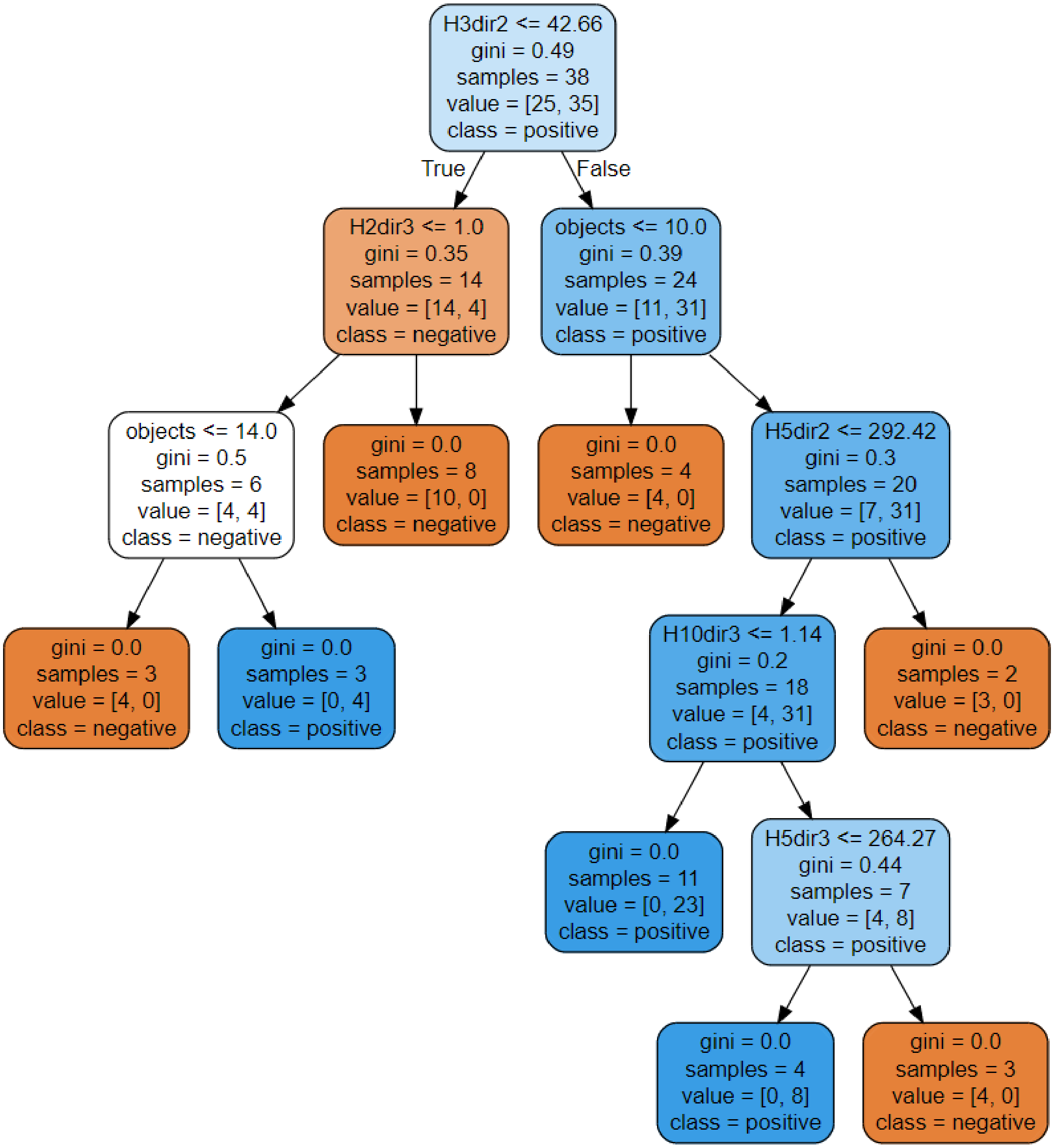
H3dir2 <= 42.66
gini = 0.49
samples = 38
value = [25, 35]
class = positive
True
False
H2dir3 <= 1.0
gini = 0.35
samples = 14
value = [14, 4]
class = negative
objects <= 10.0
gini = 0.39
samples = 24
value = [11, 31]
class = positive
objects <= 14.0
gini = 0.5
samples = 6
value = [4, 4]
class = negative
gini = 0.0
samples = 8
value = [10, 0]
class = negative
gini = 0.0
samples = 4
value = [4, 0]
class = negative
H5dir2 <= 292.42
gini = 0.3
samples = 20
value = [7, 31]
class = positive
gini = 0.0
samples = 3
value = [4, 0]
class = negative
gini = 0.0
samples = 3
value = [0, 4]
class = positive
H10dir3 <= 1.14
gini = 0.2
samples = 18
value = [4, 31]
class = positive
gini = 0.0
samples = 2
value = [3, 0]
class = negative
gini = 0.0
samples = 11
value = [0, 23]
class = positive
H5dir3 <= 264.27
gini = 0.44
samples = 7
value = [4, 8]
class = positive
gini = 0.0
samples = 4
value = [0, 8]
class = positive
gini = 0.0
samples = 3
value = [4, 0]
class = negative

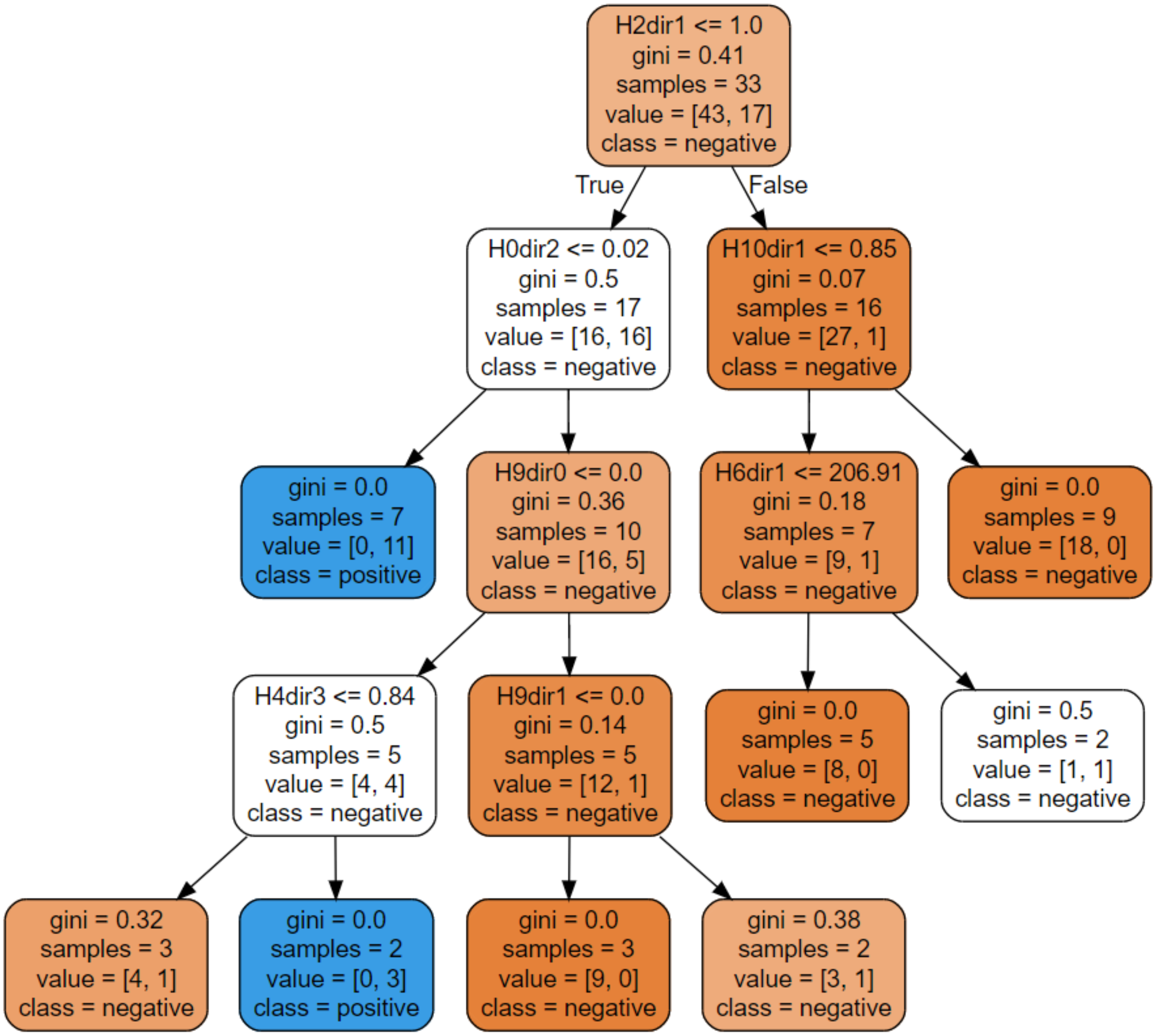
H2dir1 <= 1.0
gini = 0.41
samples = 33
value = [43, 17]
class = negative
True
False
H0dir2 <= 0.02
gini = 0.5
samples = 17
value = [16, 16]
class = negative
H10dir1 <= 0.85
gini = 0.07
samples = 16
value = [27, 1]
class = negative
gini = 0.0
samples = 7
value = [0, 11]
class = positive
H9dir0 <= 0.0
gini = 0.36
samples = 10
value = [16, 5]
class = negative
H6dir1 <= 206.91
gini = 0.18
samples = 7
value = [9, 1]
class = negative
gini = 0.0
samples = 9
value = [18, 0]
class = negative
H4dir3 <= 0.84
gini = 0.5
samples = 5
value = [4, 4]
class = negative
H9dir1 <= 0.0
gini = 0.14
samples = 5
value = [12, 1]
class = negative
gini = 0.0
samples = 5
value = [8, 0]
class = negative
gini = 0.5
samples = 2
value = [1, 1]
class = negative
gini = 0.32
samples = 3
value = [4, 1]
class = negative
gini = 0.0
samples = 2
value = [0, 3]
class = positive
gini = 0.0
samples = 3
value = [9, 0]
class = negative
gini = 0.38
samples = 2
value = [3, 1]
class = negative

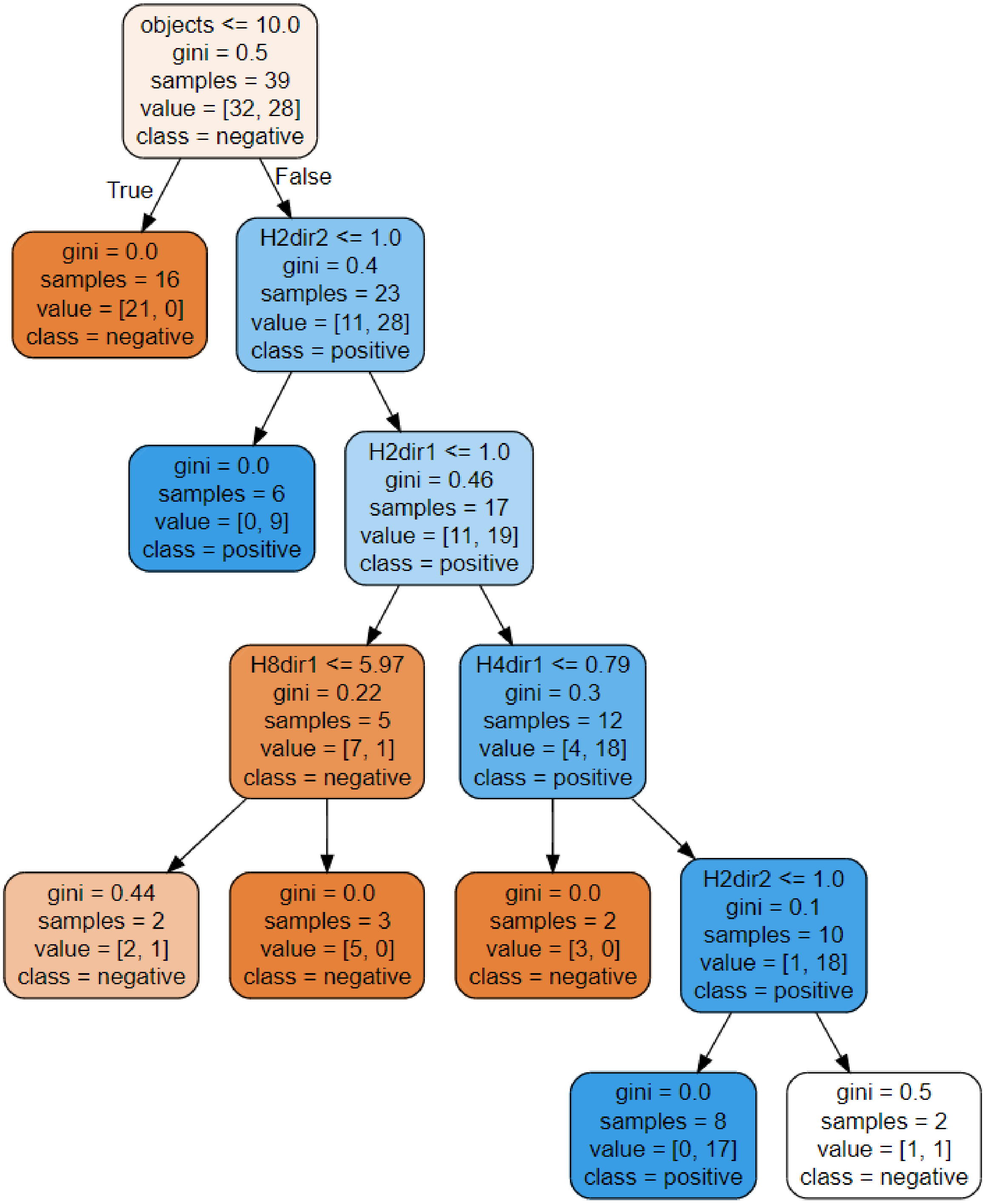
objects <= 10.0
gini = 0.5
samples = 39
value = [32, 28]
class = negative
True
False
gini = 0.0
samples = 16
value = [21, 0]
class = negative
H2dir2 <= 1.0
gini = 0.4
samples = 23
value = [11, 28]
class = positive
gini = 0.0
samples = 6
value = [0, 9]
class = positive
H2dir1 <= 1.0
gini = 0.46
samples = 17
value = [11, 19]
class = positive
H8dir1 <= 5.97
gini = 0.22
samples = 5
value = [7, 1]
class = negative
H4dir1 <= 0.79
gini = 0.3
samples = 12
value = [4, 18]
class = positive
gini = 0.44
samples = 2
value = [2, 1]
class = negative
gini = 0.0
samples = 3
value = [5, 0]
class = negative
gini = 0.0
samples = 2
value = [3, 0]
class = negative
H2dir2 <= 1.0
gini = 0.1
samples = 10
value = [1, 18]
class = positive
gini = 0.0
samples = 8
value = [0, 17]
class = positive
gini = 0.5
samples = 2
value = [1, 1]
class = negative

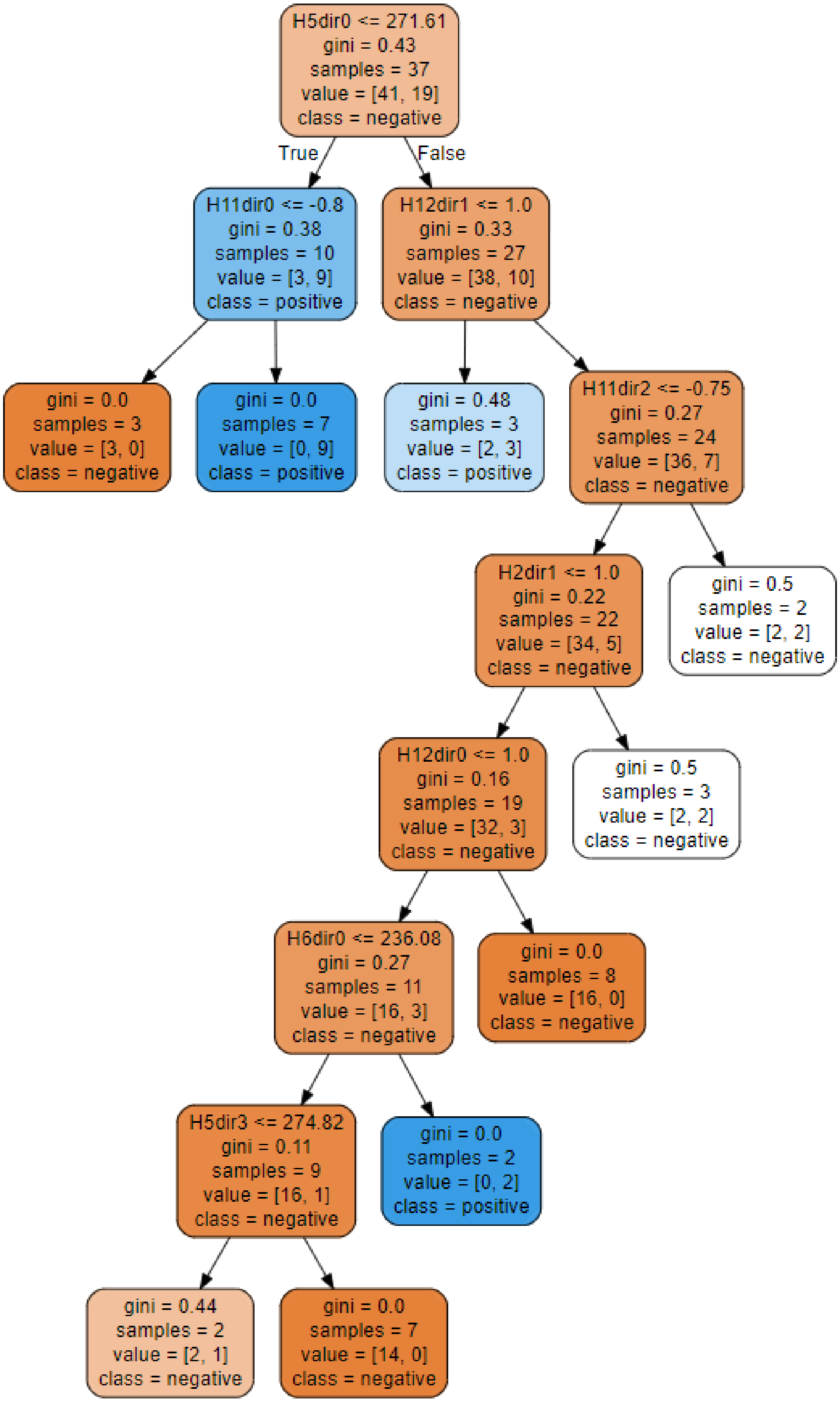
H5dir0 <= 271.61
gini = 0.43
samples = 37
value = [41, 19]
class = negative
True
False
H11dir0 <= -0.8
gini = 0.38
samples = 10
value = [3, 9]
class = positive
H12dir1 <= 1.0
gini = 0.33
samples = 27
value = [38, 10]
class = negative
gini = 0.0
samples = 3
value = [3, 0]
class = negative
gini = 0.0
samples = 7
value = [0, 9]
class = positive
gini = 0.48
samples = 3
value = [2, 3]
class = positive
H11dir2 <= -0.75
gini = 0.27
samples = 24
value = [36, 7]
class = negative
H2dir1 <= 1.0
gini = 0.22
samples = 22
value = [34, 5]
class = negative
gini = 0.5
samples = 2
value = [2, 2]
class = negative
H12dir0 <= 1.0
gini = 0.16
samples = 19
value = [32, 3]
class = negative
gini = 0.5
samples = 3
value = [2, 2]
class = negative
H6dir0 <= 236.08
gini = 0.27
samples = 11
value = [16, 3]
class = negative
gini = 0.0
samples = 8
value = [16, 0]
class = negative
H5dir3 <= 274.82
gini = 0.11
samples = 9
value = [16, 1]
class = negative
gini = 0.0
samples = 2
value = [0, 2]
class = positive
gini = 0.44
samples = 2
value = [2, 1]
class = negative
gini = 0.0
samples = 7
value = [14, 0]
class = negative

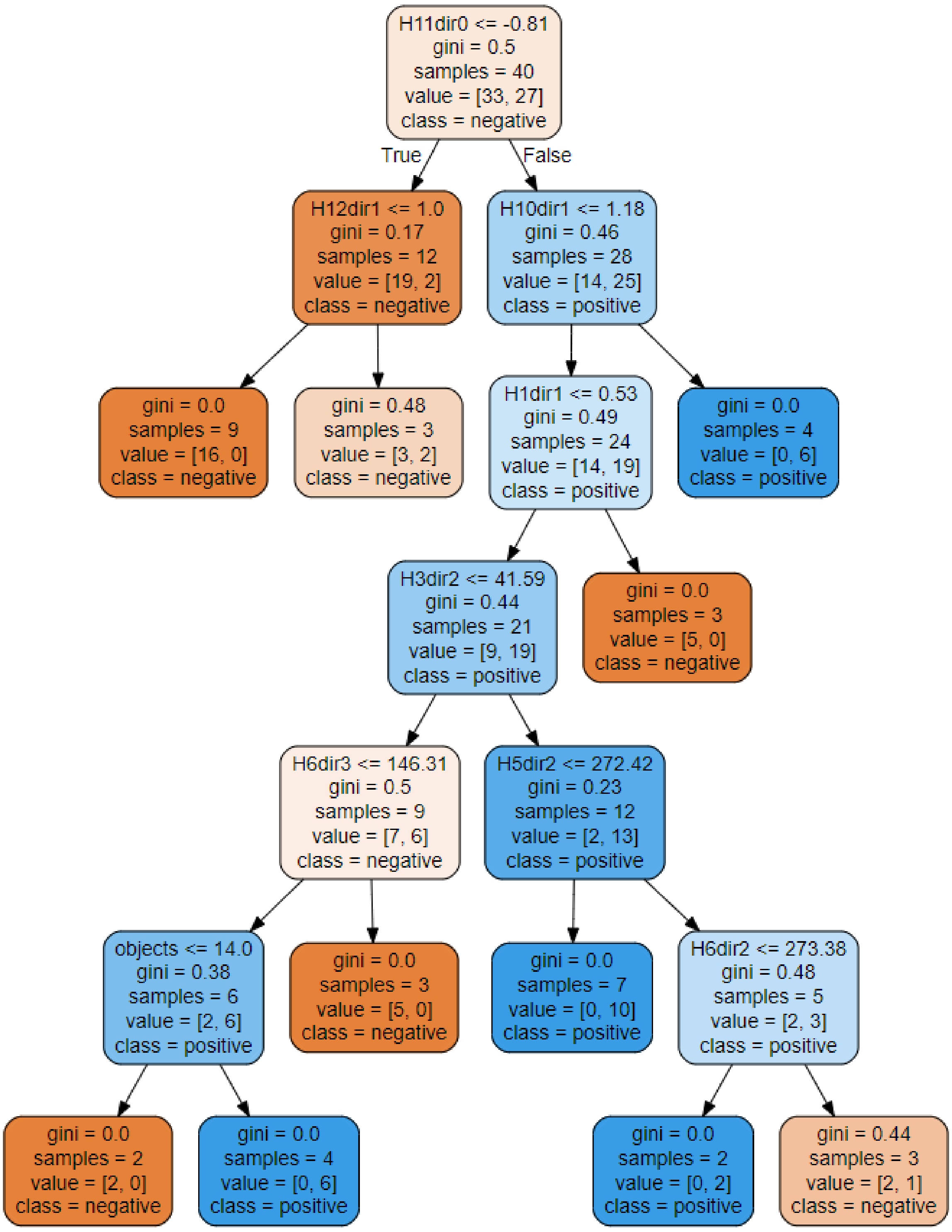
H11dir0 <= -0.81
gini = 0.5
samples = 40
value = [33, 27]
class = negative
True
False
H12dir1 <= 1.0
gini = 0.17
samples = 12
value = [19, 2]
class = negative
H10dir1 <= 1.18
gini = 0.46
samples = 28
value = [14, 25]
class = positive
gini = 0.0
samples = 9
value = [16, 0]
class = negative
gini = 0.48
samples = 3
value = [3, 2]
class = negative
H1dir1 <= 0.53
gini = 0.49
samples = 24
value = [14, 19]
class = positive
gini = 0.0
samples = 4
value = [0, 6]
class = positive
H3dir2 <= 41.59
gini = 0.44
samples = 21
value = [9, 19]
class = positive
gini = 0.0
samples = 3
value = [5, 0]
class = negative
H6dir3 <= 146.31
gini = 0.5
samples = 9
value = [7, 6]
class = negative
H5dir2 <= 272.42
gini = 0.23
samples = 12
value = [2, 13]
class = positive
objects <= 14.0
gini = 0.38
samples = 6
value = [2, 6]
class = positive
gini = 0.0
samples = 3
value = [5, 0]
class = negative
gini = 0.0
samples = 7
value = [0, 10]
class = positive
H6dir2 <= 273.38
gini = 0.48
samples = 5
value = [2, 3]
class = positive
gini = 0.0
samples = 2
value = [2, 0]
class = negative
gini = 0.0
samples = 4
value = [0, 6]
class = positive
gini = 0.0
samples = 2
value = [0, 2]
class = positive
gini = 0.44
samples = 3
value = [2, 1]
class = negative

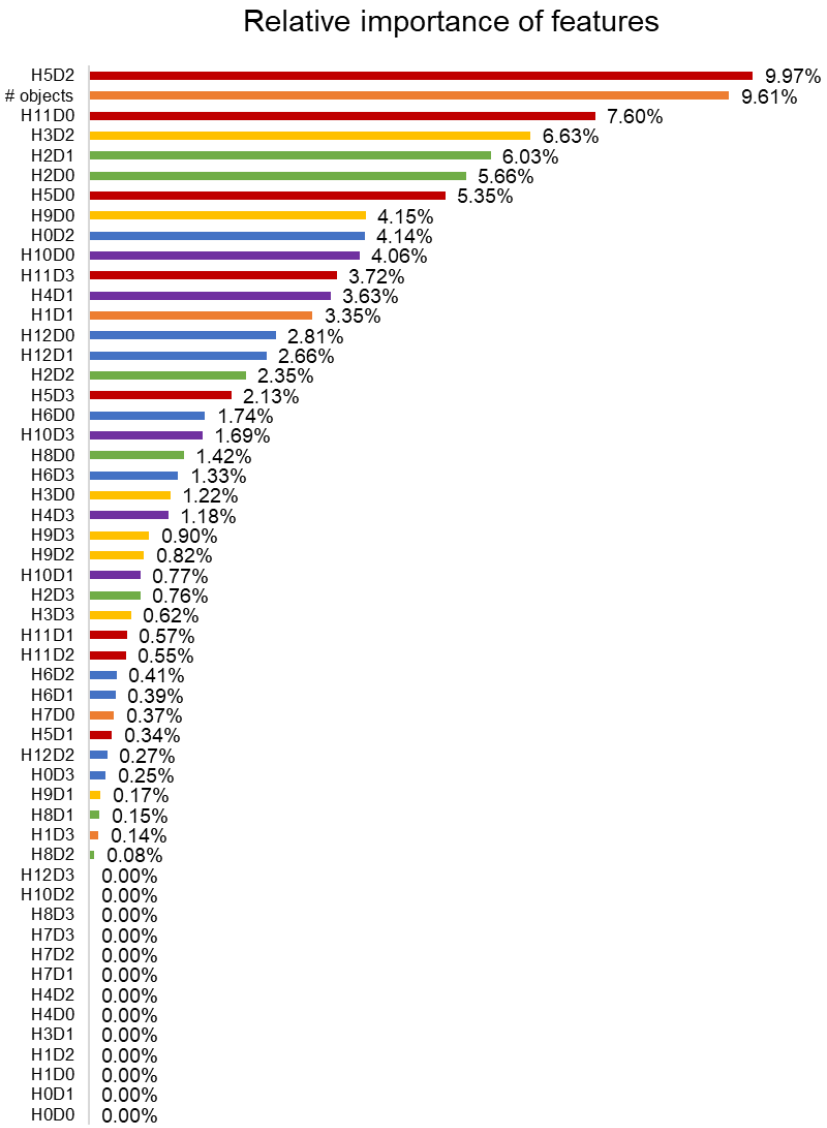

The 52 features include: # objects (detected by Laplacian-of-Gaussian algorithm) and 13 texture features computed from GLCM, each across 4 directions in the image (52 texture features).												
The 4 directions are: horizontal (0), vertical (1), left diagonal (2), and right diagonal (3).<br>The 13 features are named below:												
0	1	2	3	4	5	6	7	8	9	10	11	12
Angular second moment	Contrast	Correlation	Sum of squares: variance	Inverse difference moment	Sum average	Sum variance	Sum entropy	Entropy	Difference variance	Difference entropy	Info. measure of correlation 1	Info. measure of correlation 2
Naming convention for GLCM features listed to the right by order of predictive importance (%):<br>e.g. "H5D2" = 5th Haralick feature, 2nd direction; the sum average for GLCM across left diagonal axis.												

Figure S13. Relative importance of all features

(A) *Confusion matrix: all data (81 samples) trained on all features*

100% sensitivity<br>94% specificity<br>**98% accuracy**		*Predicted*	
		Positive	Negative
Actual	Positive	**45**	**0**
	Negative	**2**	**34**

(B) *Confusion matrix: all data (81 samples) trained on LoG only*

97% sensitivity<br>80% specificity<br>**88% accuracy**		*Predicted*	
		Positive	Negative
Actual	Positive	**35**	**1**
	Negative	**9**	**36**

(C) *Confusion matrix: all data (81 samples) trained on Haralick only*

89% sensitivity<br>94% specificity<br>**91% accuracy**		*Predicted*	
		Positive	Negative
Actual	Positive	**40**	**5**
	Negative	**2**	**34**

Figure S14. Confusion matrix of Random Forest Classifier applied to full dataset

(A) Presenting results of classifier trained on all features, collated over training and testing data results together.

(B) Presenting results of classifier based on LoG object counting feature only, with a threshold.

(C) Presenting results of classifier trained on Haralick texture features only.

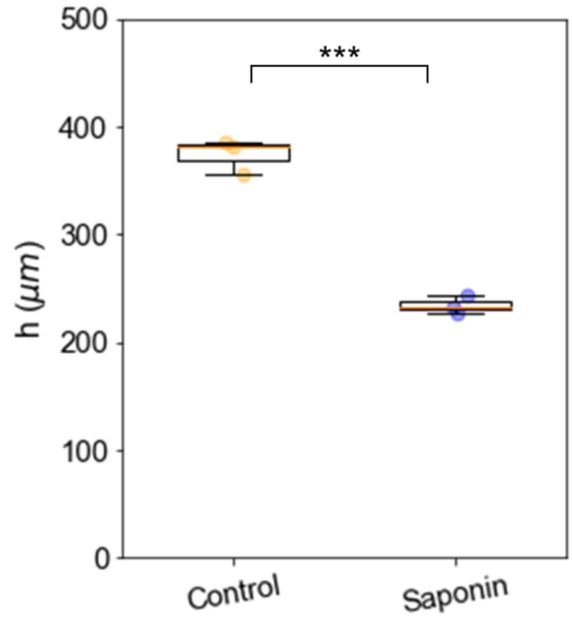

Figure S15. Levitation heights of leukocytes from whole blood:

(A) Control leukocytes (isolated by standard protocol with RBC lysis buffer, ammonium chloride) **(B)** Saponin-lysed leukocytes.
